# Supplementary material for: Increasing donor-acceptor spacing for reduced voltage loss in organic solar cells
Source: Nat Commun. 2021 Nov 18;12:6679. doi: 10.1038/s41467-021-26995-1 (PMC8602729; doi:10.1038/s41467-021-26995-1)
Supplement: Supplementary file 1 — Supplementary Information [file 41467_2021_26995_MOESM1_ESM.pdf]

Supplementary Information

Increasing Donor-Acceptor Spacing for Reduced Voltage Loss in Organic Solar Cells

Jing Wang[1], Xudong Jiang[2,3], Hongbo Wu[1], Guitao Feng[2,3], Hanyu Wu[1], Junyu Li[4], Yuanping Yi[3], Xunda Feng[1], Zaifei Ma[1], Weiwei Li[2,3,*], Koen Vandewal[5,*], Zheng Tang[1,*]

1. State Key Laboratory for Modification of Chemical Fibers and Polymer Materials, Center for Advanced Low-dimension Materials, College of Materials Science and Engineering, Donghua University, Shanghai, 201620, P. R. China
2. Beijing Advanced Innovation Center for Soft Matter Science and Engineering & State Key Laboratory of Organic-Inorganic Composites, Beijing University of Chemical Technology, Beijing 100029, P. R. China
3. Key Laboratory of Organic Solids, Institute of Chemistry, Chinese Academy of Sciences, Beijing 100190, P.R. China
4. DSM DMSC R&D Solutions, P.O. Box 18, 6160 MD Geleen, The Netherlands
5. Instituut voor Materiaalonderzoek (IMO-IMOMEC), Hasselt University, Wetenschapspark 1, BE-3590 Diepenbeek, Belgium

Emails: ztang@dhu.edu.cn; liweiwei@iccas.ac.cn; koen.vandewal@uhasselt.be

Table of Conetents

Supplementary Note 1. Materials synthesis 2

Supplementary Note 2. TEM images 9

Supplementary Note 3. GIWAXS analysis 10

Supplementary Note 4. Molecular dynamic simulations 12

Supplementary Note 5. Determination of CT state properties 15

Supplementary Note 6. Determination of $\Delta V r$ and $\Delta V n r$ 17

Supplementary Note 7. Transient photovoltage decay measurements 19

Supplementary Note 8. Photoluminescence and external quantum efficiency 22

Supplementary Note 9. JV curves of the solar cells 23

Supplementary Methods 25

Supplementary Note 1. Materials synthesis

The synthetic procedures were performed under argon atmosphere. Commercial chemicals (from Energy Chemical, Sigma-Aldrich, TCI, Innochem and JK Chemical) were used as received. The polymers **PBDB-T-NDI(C6)**, **PBDB-T-NDI(C12)**, and **PBDB-T-NDI(C18)** were synthesized by the route shown in **Supplementary Fig. 1**, compound **3a**, **3b**, **3c**, and **5** were prepared according to literature procedures (*J. Am. Chem. Soc.* 2017, 139, (51), 18647; *J. Mater. Chem. A* 2017, 5, (21), 10416; *Joule* 2019, 3, (7), 1765).

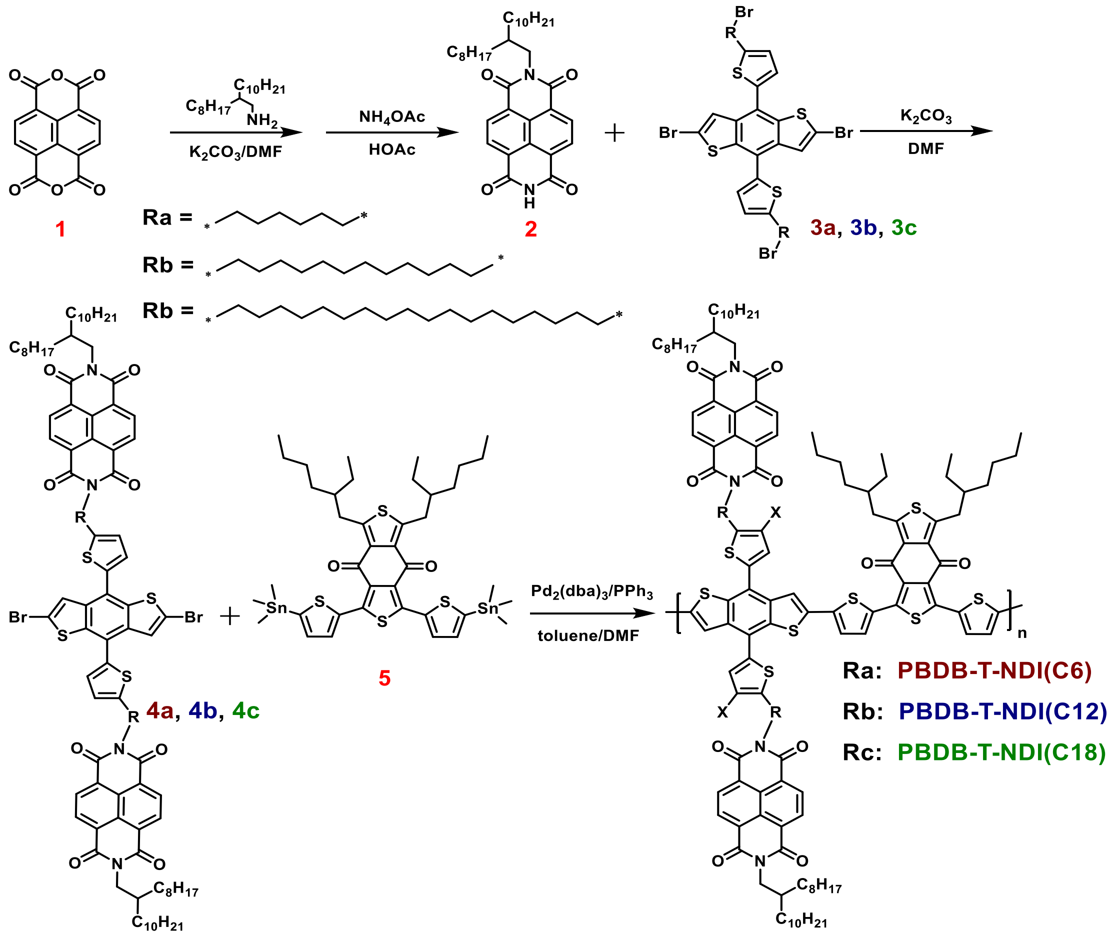

Supplementary Fig. 1. Synthetic route of the DCDA polymers.

Compound (2): Compound **(1)** (1.2 g, 4.5 mmol) was dissolved in 50 mL dry N,N-Dimethylformamide (DMF) under an inert atmosphere, 2-octyldodecan-1-amine (1.34 g, 4.5 mmol) was added. The solution was stirred at 120 °C for 12 h, the reactant was quenched by brine, then extracted by dichloromethane (100 mL), and dried over anhydrous $MgSO_4$. After removing the solvent, the crude product was dissolved in 50 mL HOAc. NH_4OAc (2.71 g, 0.045 mol) was added to the solution, then the solution was stirred at 100 °C for 4 h. The reactant was quenched by water and extracted by chloroform (100 mL), then dried over anhydrous $MgSO_4$. After removing the solvent, the crude product was purified by silica gel

chromatography (chloroform: petroleum ether, v/v = 2:1 as eluent) to obtain **2** (1.35 g, yield 55%) as white solid. ^{1}H NMR ($CDCl_3$, 400 MHz): δ (ppm) 8.81 (s, 1H), 8.75 (d, 4H), 4.13 (d, 2H), 1.99 (s, 1H), 1.22-1.37 (m, 32H), 0.83-0.88 (m, 6H). MS (MALDI-TOF) m/z: $[M]^+$, Calcd for $C_{34}H_{46}N_2O_4$: 546.3; found: 548.0.

Compound (4a): Potassium carbonate (76.0 mg, 0.55 mmol) was added into the solution of **2** (58.0 mg, 0.11 mmol) and **3a** (37.0 mg, 0.04 mmol) in DMF (30 mL). The reaction mixture was stirred at 80 °C for 24 h and then cooled to room temperature. The mixture was filtered, then washed by hot water and hot acetone to obtain **4a** (59.4 mg, yield 76%), which was a yellow solid. ^{1}H NMR (400MHz, $CDCl_3$): δ (ppm) 8.75 (t, 8H), 7.55 (s, 2H), 7.20 (d, 2H), 6.89 (d, 2H), 4.23 (t, 4H), 4.12 (d, 4H), 2.92 (t, 4H), 1.98 (m, 2H), 1.81 (s, 8H), 1.54(s, 12H), 1.37-1.22 (m, 68H), 1.81 (s, 8H), 0.85 (m, 12H). ^{13}C NMR ($CDCl_3$, 100 MHz): δ (ppm) 163.2, 162.6, 147.5, 140.2, 135.9, 135.8, 131.0, 128.0, 126.7, 126.7, 126.6, 126.5,126.0, 124.6, 122.4, 116.8, 77.3, 77.0, 76.7, 45.0, 40.8, 36.6, 31.9, 31.9, 31.7, 31.4, 31.1, 30.0, 29.6, 29.6, 29.5, 29.3, 29.3, 28.8, 28.0, 26.8, 26.4, 22.7, 22.6, 14.4. HRMS(MALDI-TOF) m/z: $[M]^+$, Calcd for $C_{98}H_{120}Br_2N_4O_8S_4$: 1766.63612; found: 1766.63893.

Compound (4b): Potassium carbonate (48.3 mg, 0.35 mmol) was added into the solution of **2** (40.4 mg, 0.07 mmol) and **3b** (25.0 mg, 0.02 mmol) in DMF (30 mL). The reaction mixture was stirred at 80 °C for 24 h and then cooled to room temperature. Then, the mixture was filtered, washed by hot water and hot acetone to obtain **4b** (30.6 mg, yield 79%), which is a yellow solid. ^{1}H NMR (400MHz, $CDCl_3$): δ (ppm) ^{1}H NMR (400MHz, $CDCl_3$): δ (ppm) 8.75 (s, 8H), 7.56 (s, 2H), 7.20 (d, 2H), 6.89 (d, 2H), 4.20 (t, 4H), 4.12 (d, 4H), 2.90 (t, 4H), 1.98 (m, 2H), 1.76 (t, 8H), 1.43-1.22 (m, 104H), 0.85 (m, 12H). ^{13}C NMR ($CDCl_3$, 100 MHz): δ (ppm) 163.2, 162.8, 147.8, 140.2, 135.9, 135.7, 131.0, 130.9, 128.0, 126.7, 126.6, 126.0, 124.5, 122.4, 116.7, 77.3, 77.0, 76.7, 45.0, 41.0, 36.6, 31.9, 31.9, 31.7, 31.6, 30.2, 30.0, 29.6, 29.6, 29.5, 29.5, 29.3, 29.3, 29.2, 28.1, 27.1, 26.4, 22.7, 22.6, 14.4. HRMS(MALDI-TOF) m/z: $[M]^+$, Calcd for $C_{110}H_{144}Br_2N_4O_8S_4$: 1934.82392; found:1934.82334.

Compound (4c): Potassium carbonate (110.4 mg, 0.80 mmol) was added to the solution of **2** (135.0 mg, 0.25 mmol) and **3c** (83.1 mg, 0.07 mmol) in DMF (50 mL). The reaction mixture was stirred at 80 °C for 24 h and then cooled to room temperature. Then, the mixture was filtered, washed by hot water and hot acetone to obtain **4c** (184.0 mg, yield 81%) as a gray solid. ^{1}H NMR (400MHz, $CDCl_3$): δ (ppm) ^{1}H NMR (400MHz, $CDCl_3$): δ (ppm). 8.75 (s, 8H), 7.56 (s, 2H), 7.21 (d, 2H), 6.89 (d, 2H), 4.18 (t, 4H), 4.13 (d, 4H), 2.89 (t, 4H), 1.98 (m, 2H), 1.78-1.73 (m, 8H), 1.54-1.21 (m, 128H), 0.85 (m, 12H). ^{13}C NMR ($CDCl_3$, 100 MHz): δ (ppm) 163.2, 162.8, 147.8, 140.2, 135.9, 135.7, 131.0, 130.9, 127.9, 126.7, 126.6, 126.0, 124.5, 122.4, 116.7, 45.0, 36.6, 31.9, 31.9, 31.6, 31.6, 30.2, 30.0, 29.7, 29.6, 29.6, 29.5, 29.3, 29.3, 29.2, 28.1, 27.1, 26.4, 22.7, 14.4. HRMS (MALDI-TOF) m/z: $[M]^+$, Calcd for $C_{122}H_{168}Br_2N_4O_8S_4$: 2103.011725; found:2103.012869.

PBDB-T-NDI(C6): To the degassed solution of monomer **4a** (33.98 mg, 19.20 μmol), **5** (17.94 mg, 19.20 μmol) in toluene (3 mL) and DMF (0.3 mL), $Pd_2(dba)_3$ (0.53 mg, 0.58 μmol) and PPh_3 (0.60 mg, 2.30 μmol) were added. The mixture was stirred at 115 °C for 36 h, then precipitated in methanol and filtered through a Soxhlet thimble. The polymer was extracted with acetone, hexane, dichloromethane, and chloroform. Then, the solvent was evaporated, the polymer was precipitated in acetone and collected by filtering using a 0.45 μm PTFE membrane filter and drying in a vacuum oven. **PBDB-T-NDI(C6)** (37.5 mg, 88.0%) is a dark solid. GPC (*o*-DCB, 140 °C): M_n = 86.0 kg mol^{-1}, M_w = 129.9 kg mol^{-1} and PDI = 1.51.

PBDB-T-NDI(C12): To the degassed solution of monomer **4b** (31.34 mg, 16.17 μmol), **5** (17.80 mg, 16.17 μmol) in toluene (2.5 mL) and DMF (0.25 mL), $Pd_2(dba)_3$ (0.44 mg, 0.49 μmol) and PPh_3 (0.51 mg, 1.94 μmol) were added. The mixture was stirred at 115 °C for 36 h, then precipitated in methanol and filtered through a Soxhlet thimble. The polymer was extracted with acetone, hexane, dichloromethane, and chloroform. Then, the solvent was evaporated, the polymer was precipitated in acetone and collected by filtering using a 0.45 μm PTFE membrane filter and drying in a vacuum oven. **PBDB-T-NDI(C12)** (33.8 mg, 85.0%) is a dark solid. GPC (*o*-DCB, 140 °C): M_n = 90.9 kg mol^{-1}, M_w = 132.1 kg mol^{-1} and PDI = 1.45.

PBDB-T-NDI(C18): To a degassed solution of monomer **4c** (35.51 mg, 16.85 μmol), **5** (15.75 mg, 16.85 μmol) in toluene (2.5 mL) and DMF (0.25 mL), $Pd_2(dba)_3$ (0.46 mg, 0.51 μmol) and PPh_3 (0.53 mg, 2.02 μmol) were added. The mixture was stirred at 115 °C for 36 h, then precipitated in methanol and filtered through a Soxhlet thimble. The polymer was extracted with acetone, hexane, dichloromethane, and chloroform. Then, the solvent was evaporated, the polymer was precipitated in acetone and collected by filtering using a 0.45 μm PTFE membrane filter and drying in a vacuum oven. **PBDB-T-NDI(C18)** (38.4 mg, 88.0%) is a dark solid. GPC (*o*-DCB, 140 °C): M_n = 192.1 kg mol^{-1}, M_w = 224.5 kg mol^{-1} and PDI = 1.27.

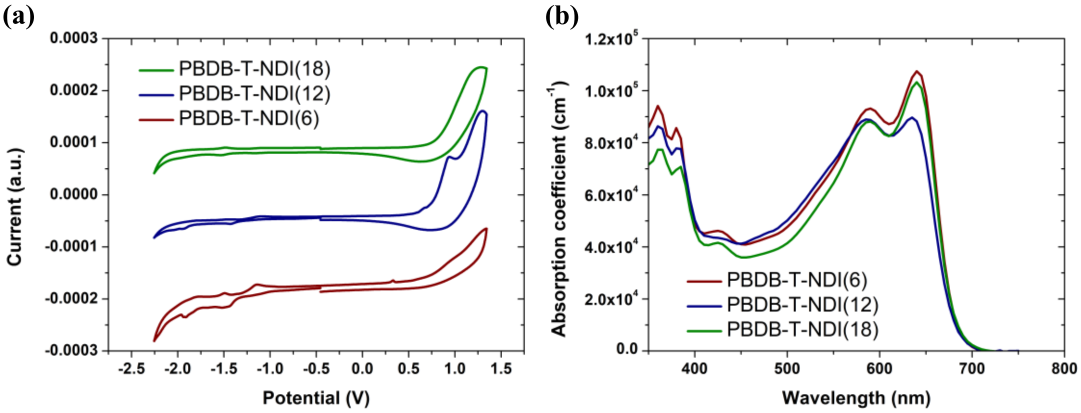

Supplementary Fig. 2. a) Cyclic voltammogram of the thin films based on the DCDA polymers, potential vs. Fc/Fc+. **b)** Absorption coefficient spectra of the DCDA polymers.

Supplementary Table 1. Molecular weight and optical properties of the DCDA Polymers.

Polymer	M_n [a] (kg/mol)	M_w [a] (kg/mol)	PDI	E_{HOMO} (eV)	E_{LUMO} (eV)	E_g^{CV} (eV)
**PBDB-T-NDI(C6)**	86.0	129.9	1.51	-5.51	-3.70	1.81
**PBDB-T-NDI(C12)**	90.9	132.1	1.45	-5.54	-3.76	1.78
**PBDB-T-NDI(C18)**	192.1	244.5	1.27	-5.56	-3.75	1.81

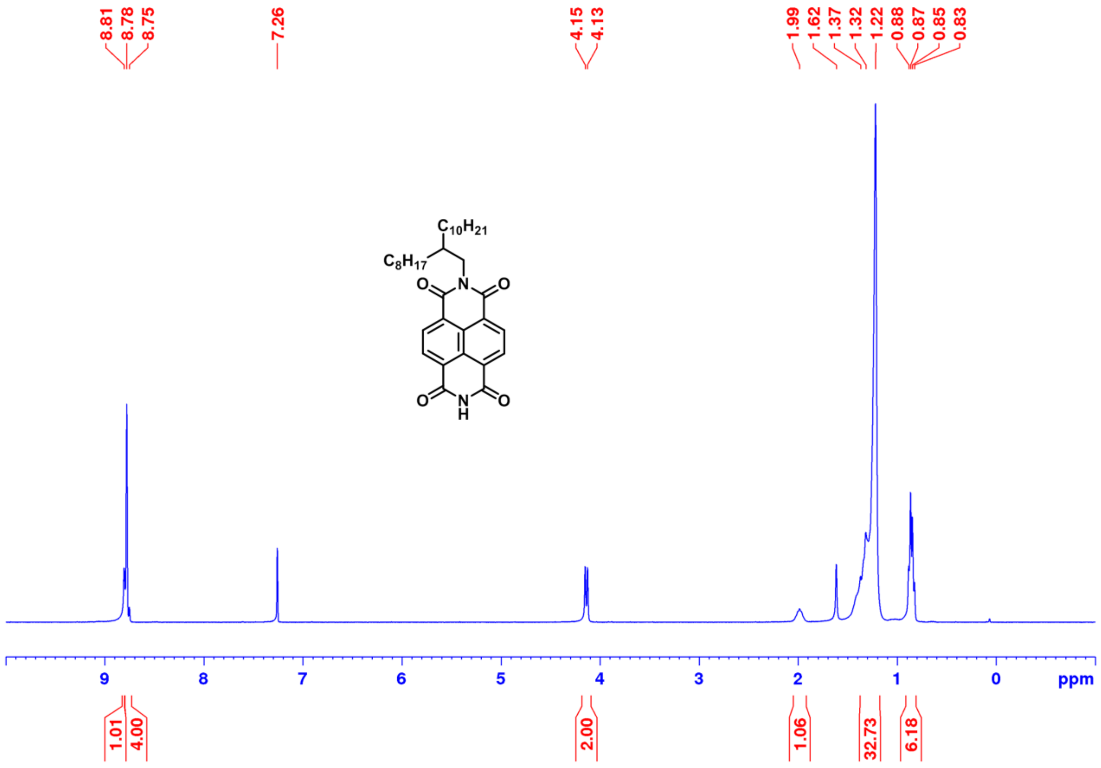

Supplementary Fig. 3. ^{1}H-NMR of the compound **2** recorded in $CDCl_3$.

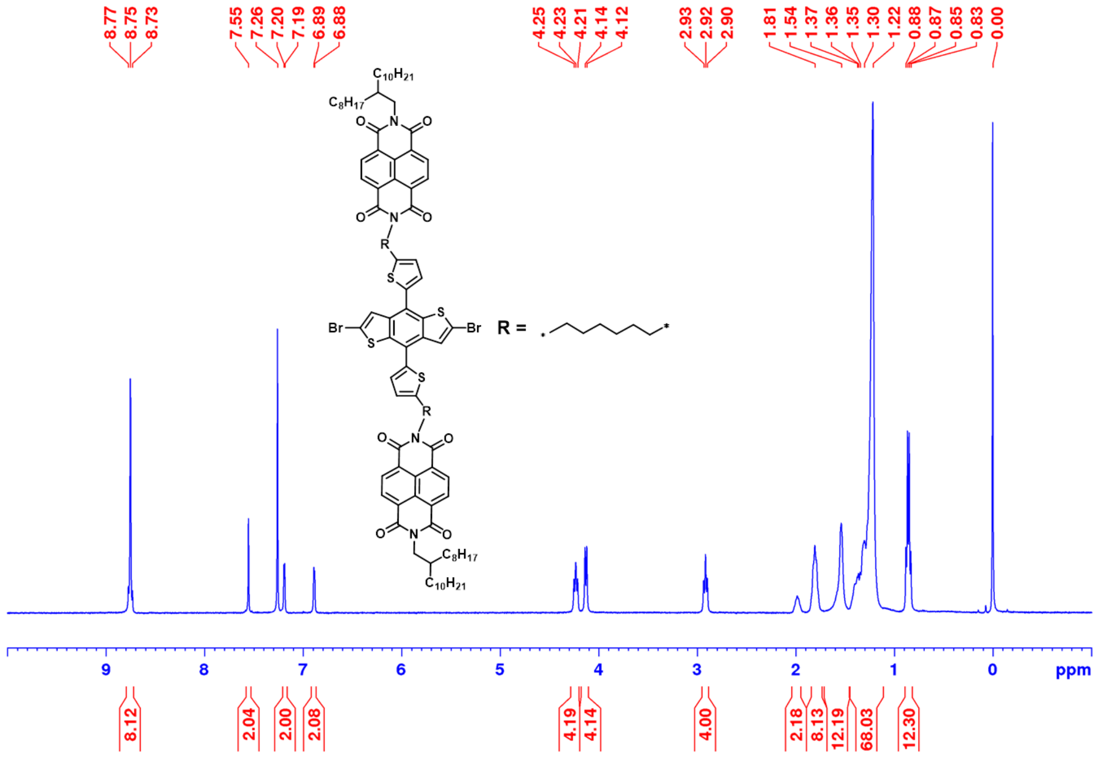

Supplementary Fig. 4. ^{1}H-NMR of the compound **4a** recorded in $CDCl_3$.

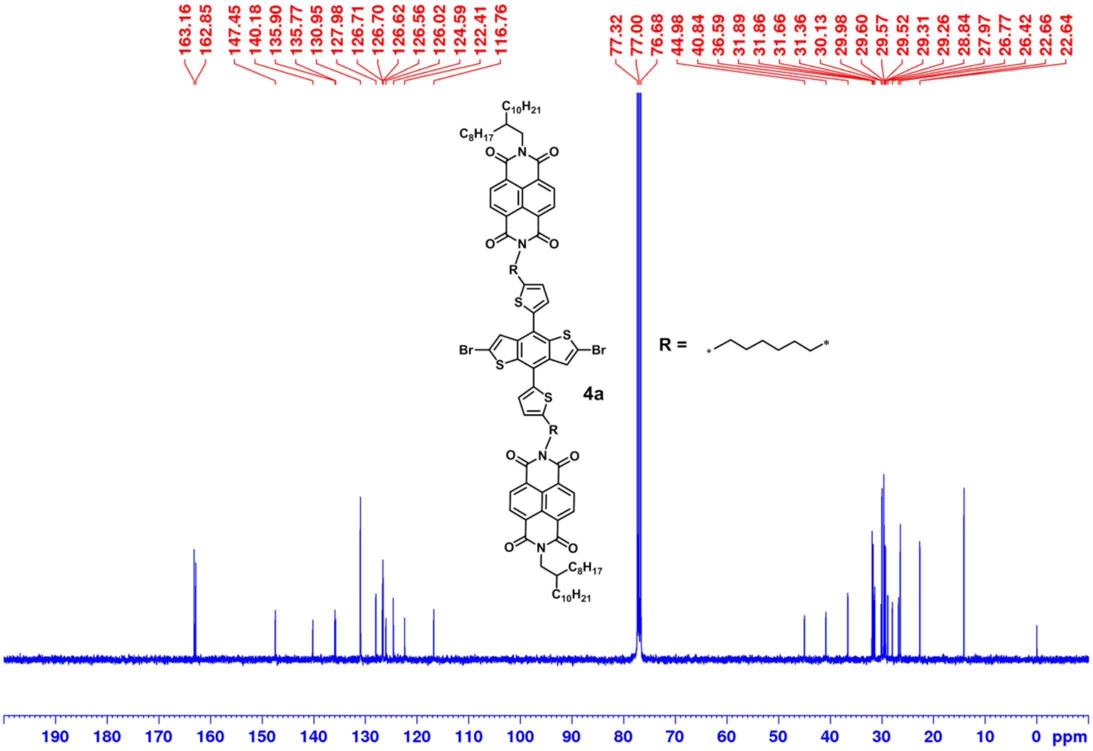

Supplementary Fig. 5. ^{13}C-NMR of the compound **4a** recorded in $CDCl_3$.

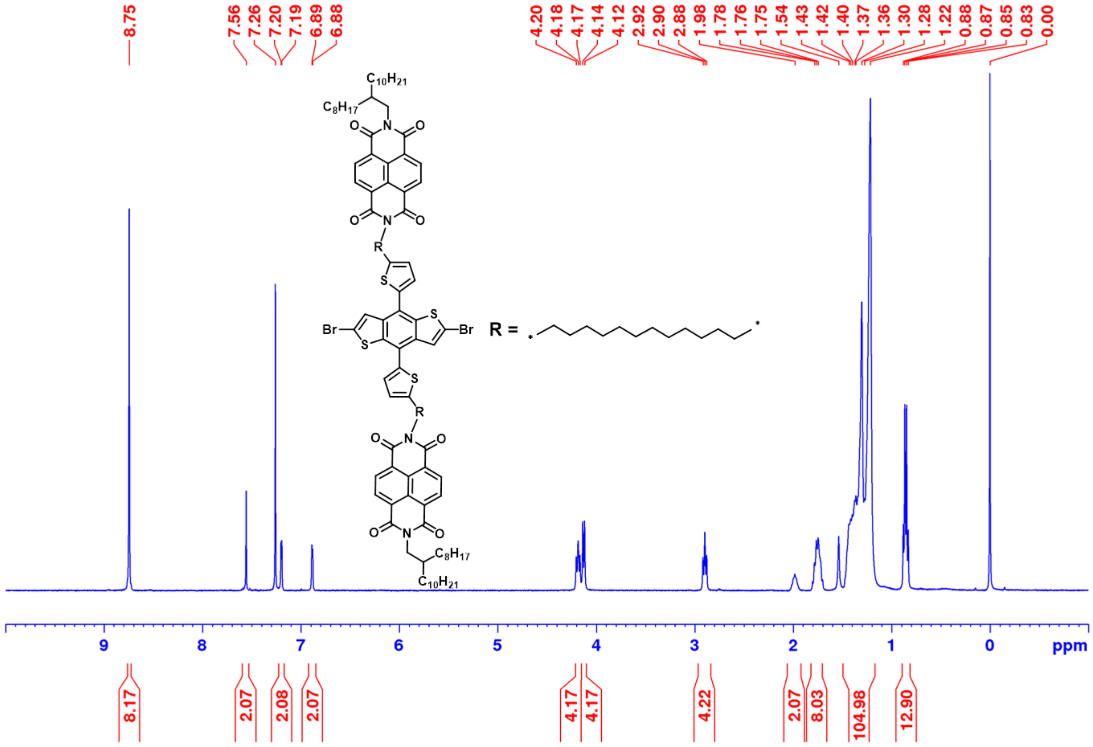

Supplementary Fig. 6. ^{1}H-NMR of the compound **4b** recorded in $CDCl_3$.

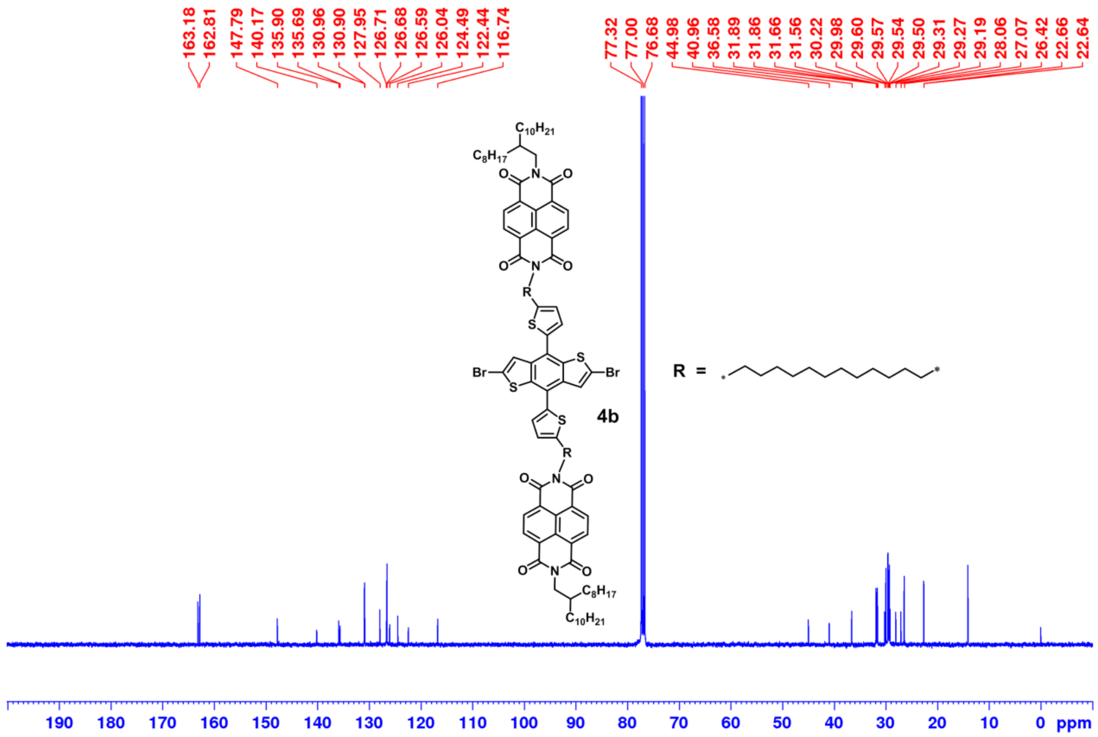

Supplementary Fig. 7. ^{13}C-NMR of the compound **4b** recorded in $CDCl_3$.

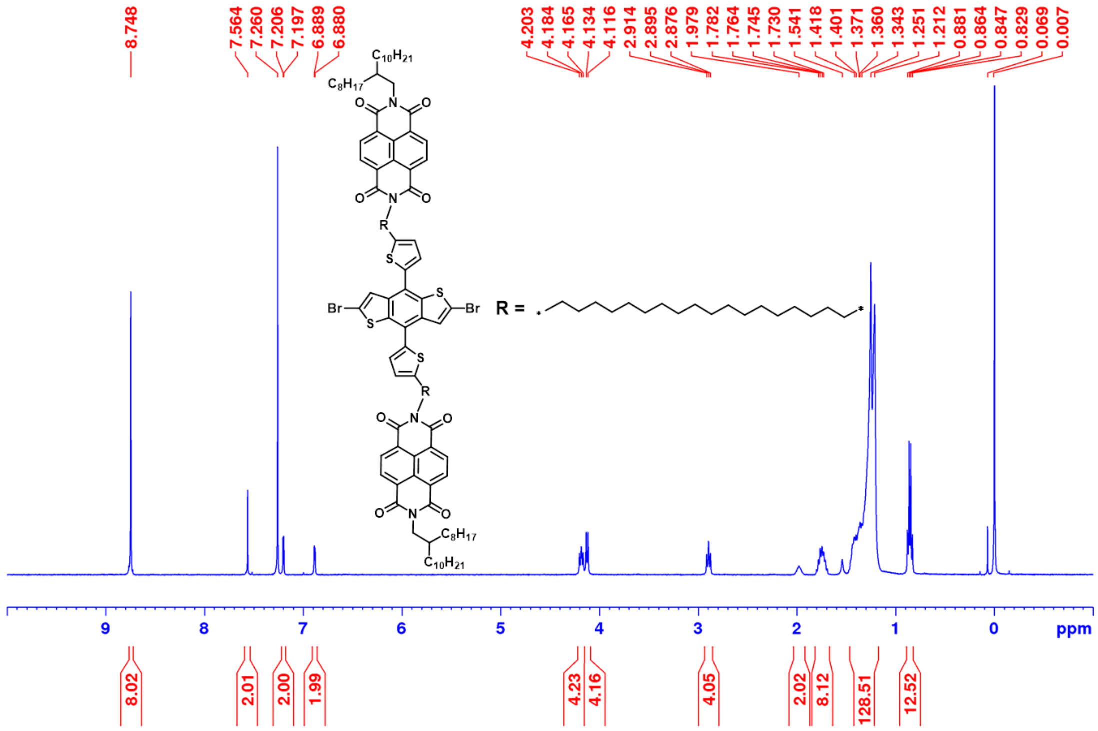

Supplementary Fig. 8. ^{1}H-NMR of the compound **4c** recorded in $CDCl_3$.

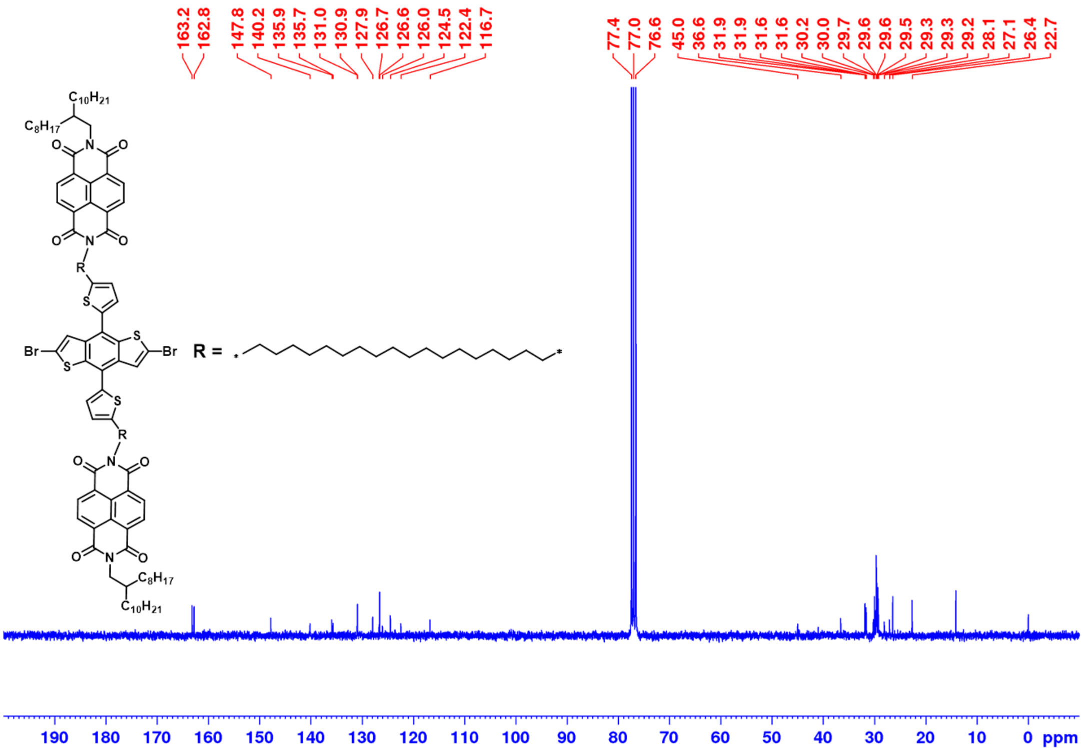

Supplementary Fig. 9. ^{13}C-NMR of the compound **4c** recorded in $CDCl_3$.

Supplementary Note 2. TEM images

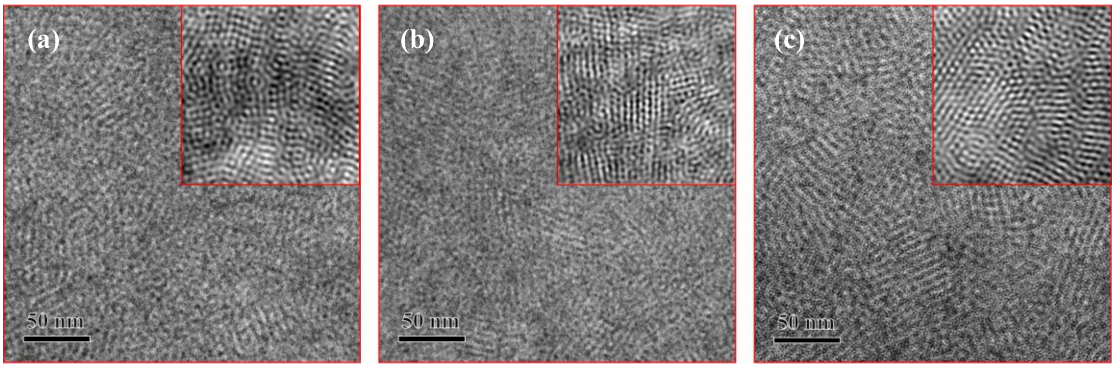

Supplementary Fig. 10. TEM images of the thin films of **a)** PBDB-T-NDI(C6), **b)** PBDB-T-NDI(C12), and **c)** PBDB-T-NDI(C18). Insets are the images obtained by inverse fast Fourier transforms (FFTs) of the filtered FFTs of the original one using ImageJ.

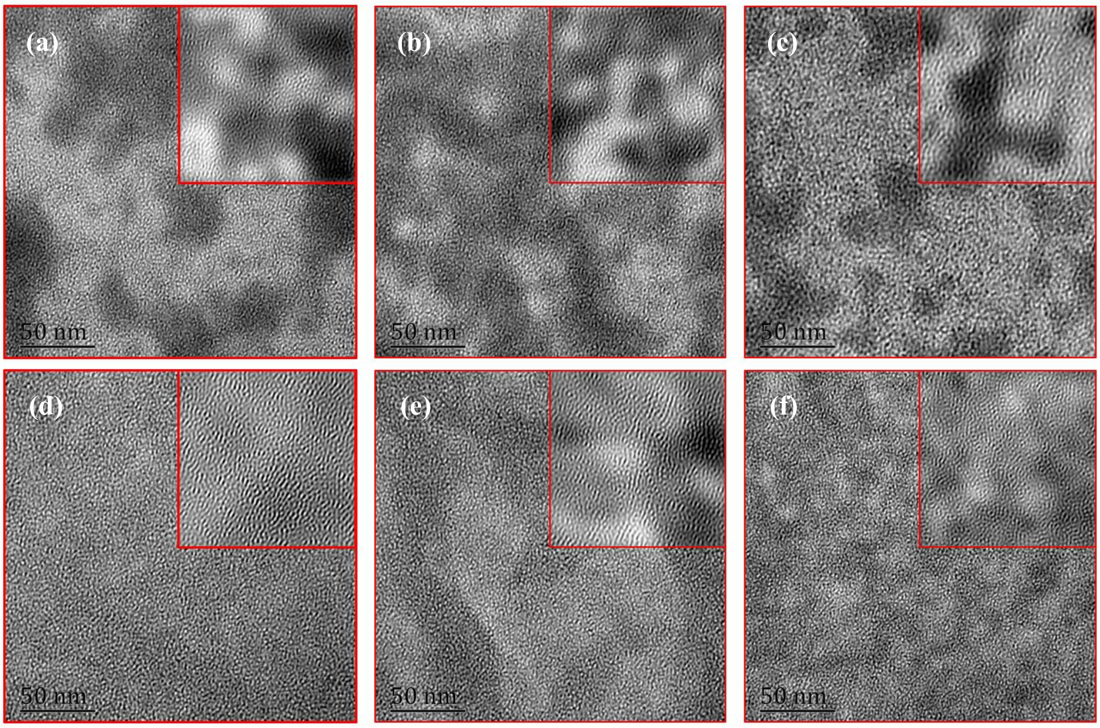

Supplementary Fig. 11. TEM images of the BHJ thin films of **a)** PBDB-T:ITIC, **b)** PBDB-T:IT4F, **c)** PBDB-T:Y6, **d)** PBDB-T(OD):ITIC, **e)** PBDB-T(OD):IT4F, and **f)** PBDB-T:Y6(OD). Insets are the images obtained by inverse fast Fourier transforms (FFTs) of the filtered FFTs of the original one using ImageJ.

Supplementary Note 3. GIWAXS analysis

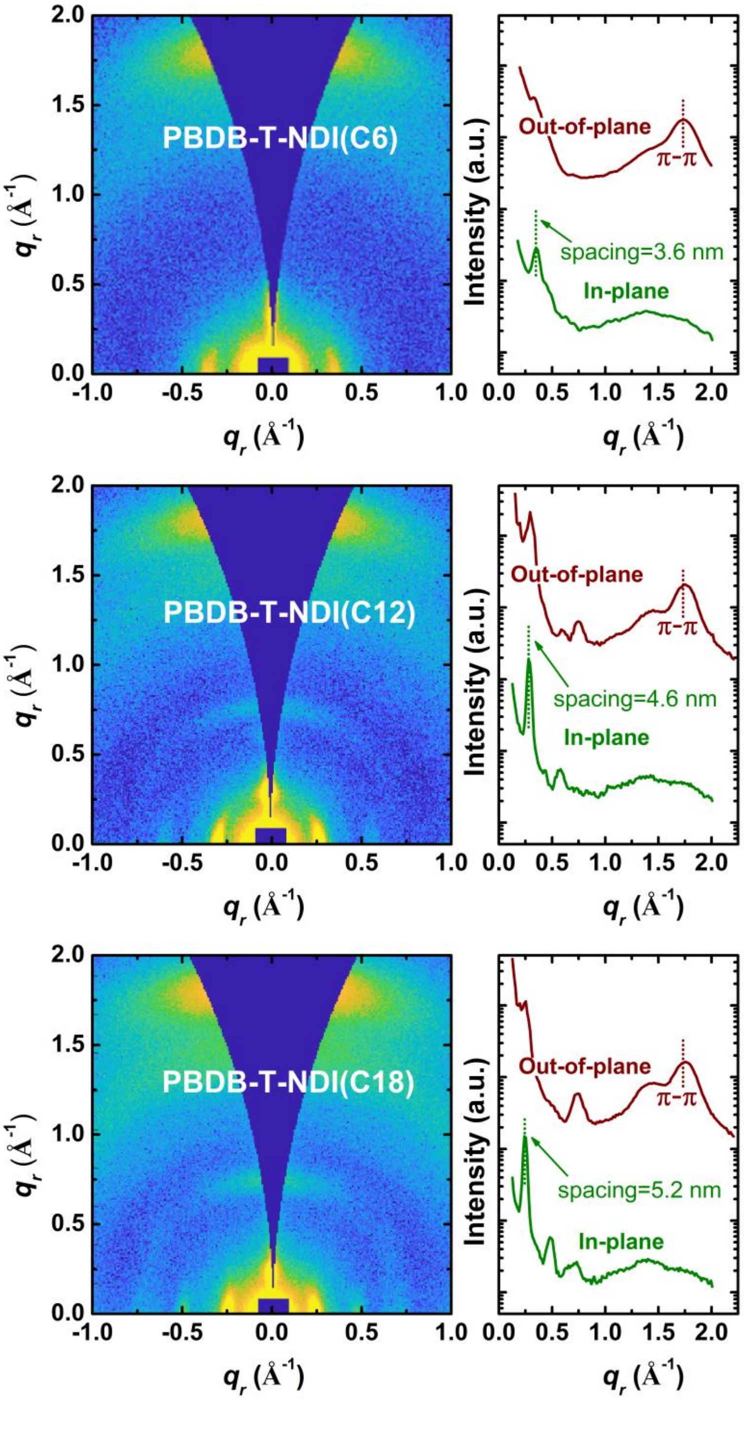

Supplementary Fig. 12. Grazing incident wide angle diffraction (GIWAXS) patterns of the DCDA polymers with different sizes of alkyl chain linkers. The *d*-spacing values are determined using the Scherrer equation.

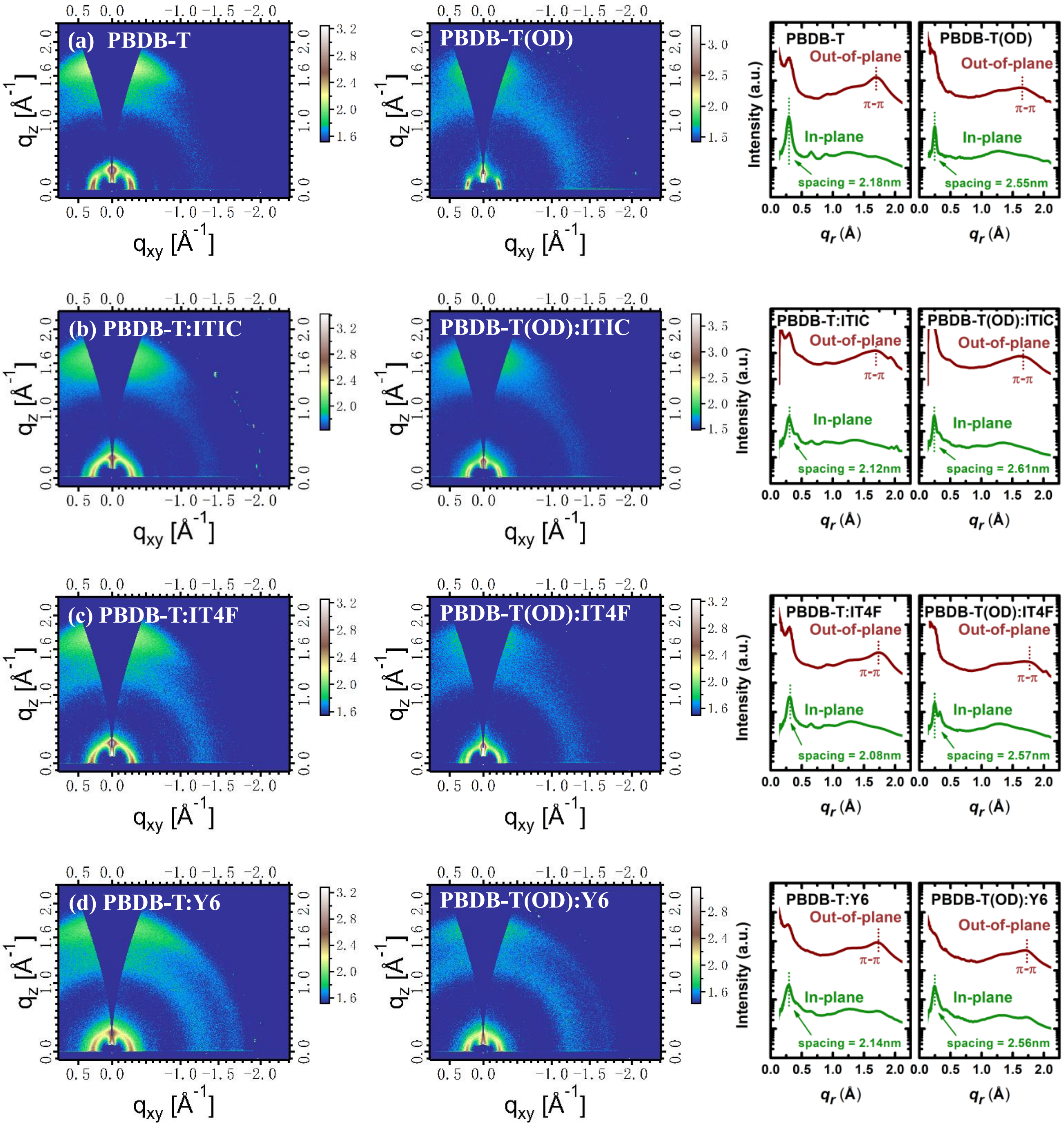

Supplementary Fig. 13. GIWAXS patterns of **a)** the pristine thin films of PBDB-T and PBDB-T(OD), and the BHJ systems based on PBDB-T and PBDB-T(OD) mixed with **b)** ITIC, **c)** IT-4F, and **d)** Y6. The *d*-spacing values are determined using the Scherrer equation. From the GIWAXS patterns of the pristine thin films of PBDB-T and PBDB-T(OD), lamellar structures with a vertical orientation are found. The equatorial scattering is at q_x = 0.30 Å^{-1} for the film of PBDB-T, which is considerably larger than that of PBDB-T(OD) (q_x = 0.25 Å^{-1}), suggesting that the distance between the adjacent layers in the lamella increases with the increasing size of the side chains. The GIWAXS patterns of the films of the BHJ systems based on different acceptors are very similar to that of the pristine donor polymers, showing lamellar structures. This suggests that the scattering signals mainly originate from the crystalline donor phases in the BHJ thin films. Also, we note that the distance between the layers in the lamella of the film of the BHJ system increases with the increasing size of the side chains. The GIWAXS results indicate that the side chains of the donor do play a crucial role in determining the arrangement of the molecules in the BHJ systems, in particular, the spacing between the molecules.

Supplementary Note 4. Molecular dynamic simulations

Molecular dynamics (MD) simulations were performed using the Materials Studio 6.0 package. The force field used was Compass force field.

The active systems based on the DCDA polymers were simulated by cubic cells, and in each cell, there were 6 polymers, and each polymer consists of 10 repeating units. The initial simulations were carried out by randomly placing the polymers in the cells with a very low density (0.1 g cm^{-3}). The corresponding construction process was done using the Amorphous Cell package in Materials Studio, and the ring spearing and close contacts were examined. The sizes of the initial cells for PBDB-T-NDI(C6) and PBDB-T-NDI(C18) were 130.3 × 130.3 × 130.3 $Å^3$ and 136.6 × 136.6 × 136.6 $Å^3$, respectively. Then, subsequent MD simulations were performed with the NPT ensemble from 650 K (500 ps) to 473 K (500 ps), and then to 300 K (1 ns) at a pressure of 1 atmosphere (473 K is the temperature used for annealing the active layers of the solar cells). The cooling from 650 K to 473 K was done in three successive NPT simulations (600 K for 100 ps, 550 K for 100 ps, 500 K for 100 ps). After the simulation of the annealing process at the 473 K, the cooling from 473 K to 300 K was done in four successive NPT simulations (450 K for 100 ps, 400 K for 100 ps, 350 K for 100 ps, and 300K for 1 ns). The Nose-Hoover thermostat/barostat was used for controlling the temperature and the pressure. The time step for the NPT simulations was 1 fs, and the cutoff for the summation of van der Waals interactions was 12.5 Å. After equilibrium, the sizes of the cells were 58.12 × 58.12 × 58.12 $Å^3$ and 61.59 × 61.59 × 61.59 $Å^3$ for PBDB-T-NDI(C6) and PBDB-T-NDI(C18), respectively.

The BHJ active systems based on PBDB-T and PBDB-T(OD) mixed with different acceptors were simulated by cubic cells, and in each cell, there were 5 polymers and each polymer consists of 10 repeating units. The numbers of the acceptor molecules put in the cells were determined from the donor:acceptor weight ratios used for fabricating the corresponding solar cells. The initial simulations were carried out by randomly placing the polymers and the acceptor molecules in the cells with a very low density (0.1 g cm^{-3}). The corresponding construction process was done using Amorphous Cell package in Materials Studio, and the ring spearing and close contacts were examined. Then, subsequent MD simulations were performed with the NPT ensemble from 650 K (500 ps) to 393 K (500 ps), and then to 300 K (1 ns) at a pressure of 1 atmosphere (393 K is the temperature used for annealing the active layers of the solar cells). The cooling from 650 K to 393 K was done in five successive NPT simulations (600 K for 100 ps, 550 K for 100 ps, 500 K for 100 ps, 450 K for 100 ps and 400K for 100ps). After the simulation of the annealing process at the 393 K, the cooling from 393 K to 300 K was done in three successive NPT simulations (393 K for 500 ps, 350K for 100 ps and 300K for 1 ns). The Nose-Hoover thermostat/barostat was used for controlling the temperature and the pressure. The time step for the NPT simulations was 1 fs, and the cutoff for the summation of van der Waals interactions was 12.5 Å.

The simulation results for the solar cells based on the DCDA polymers are given in **Supplementary Fig. 14**, and the results for the BHJ solar cells based on PBDB-T and PBDB-T(OD) mixed with ITIC and Y6 are shown below in **Supplementary Fig. 15** and **Supplementary Fig. 16**, respectively. The results for the BHJ solar cells based on PBDB-T:IT4F and PBDB-T(OD):IT4F are shown in Fig. 3.

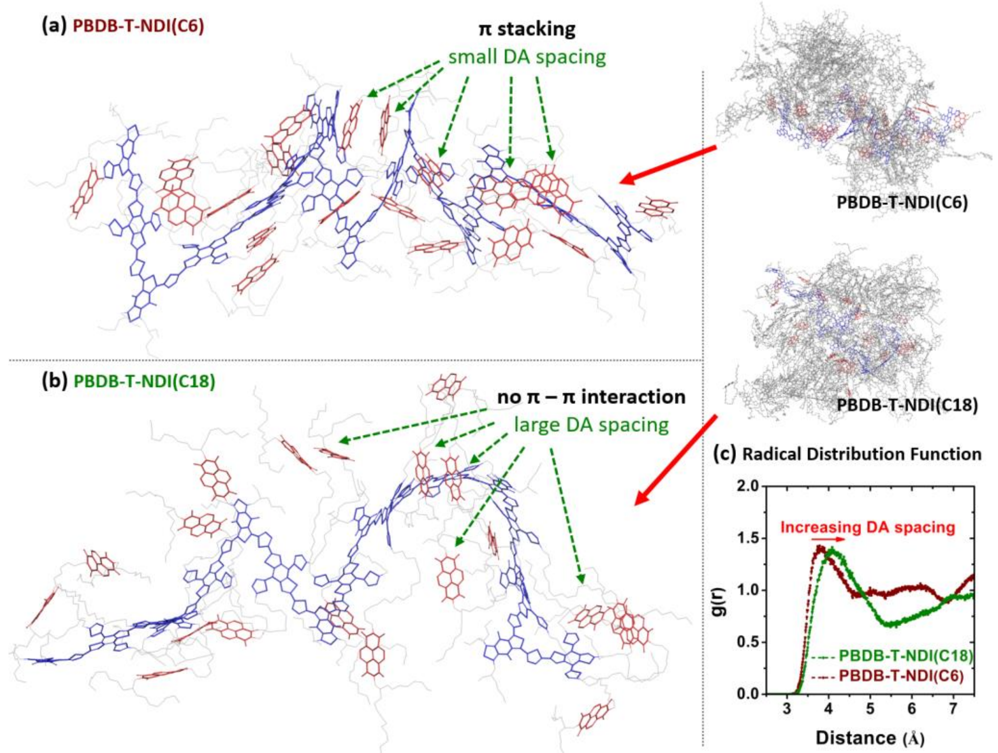

Supplementary Fig. 14. MD simulation results for **a)** PBDB-T-NDI(C6) and **b)** PBDB-T-NDI(C18). Close parallel π stacking between NDI and the polymer backbone is predicted for PBDB-T-NDI(C6), while the distance between NDI and the polymer backbone is considerably larger for PBDB-T-NDI(C18), due to the large space around the polymer backbone occupied by the alkyl chains, preventing π stacking between NDI and the backbone. **c)** RDF of the distances between NDI and the polymer backbone for PBDB-T-NDI(C6) and PBDB-T-NDI(C18).

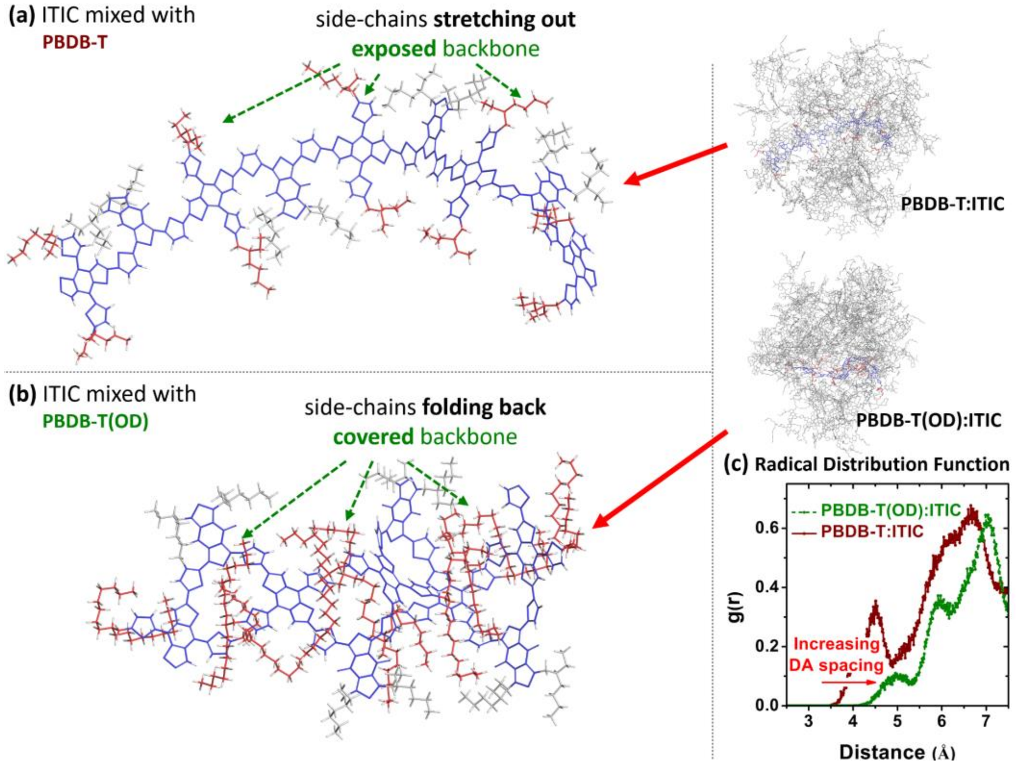

Supplementary Fig. 15. MD simulation results for **a)** PBDB-T and **b)** PBDB-T(OD) blended with ITIC. **c)** RDF of the distances between the donor backbones and ITIC.

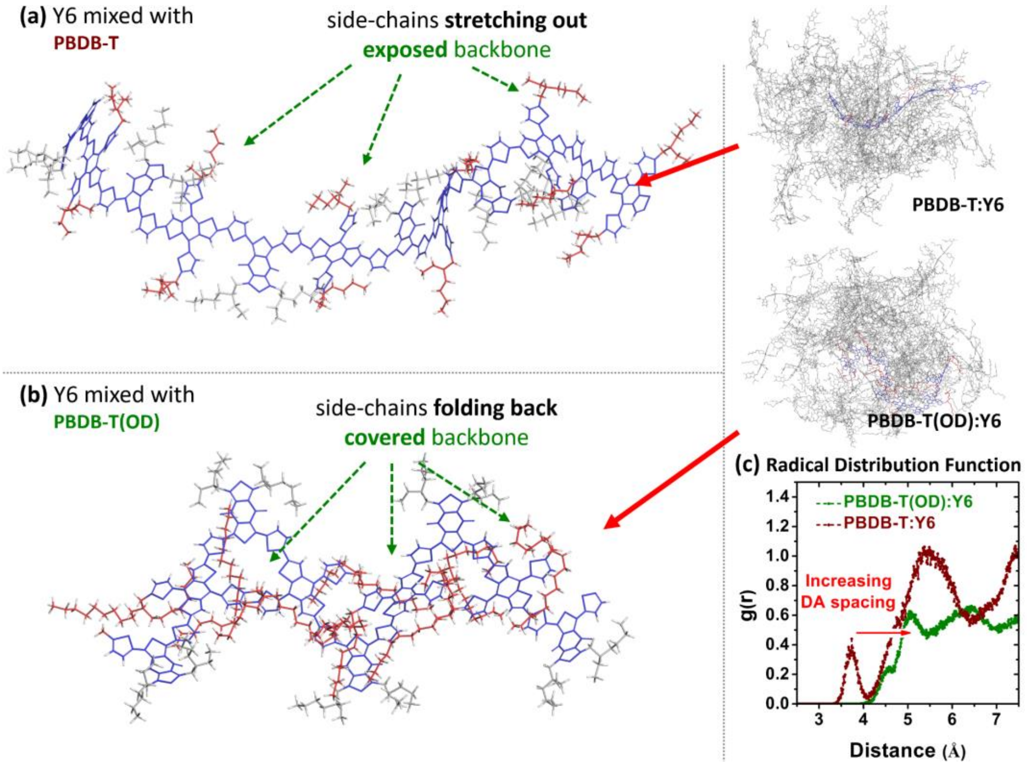

Supplementary Fig. 16. MD simulation results for **a)** PBDB-T and **b)** PBDB-T(OD) blended with Y6. **c)** RDF of the distances between the donor backbones and Y6.

Supplementary Note 5. Determination of CT state properties

The CT state properties, including E_{CT} and the reorganization energy (λ), are determined by analyzing the tails of the sensitive EQE spectra of the solar cells, measured by a sensitive EQE setup. The measurement setup consists of a halogen lamp, a monochromator, a current amplifier and a lock-in amplifier. A set of long pass filters are used to make sure that the higher order wavelengths from the monochromator are completely cut off, and light reaching the device under test is truly monochromatic. Then, the electroluminescence (EL) spectra of the solar cells are measured using a small injection current (1 mA), a spectrometer, and cooled Si and InGaAs detectors (-70 °C), and the spectra are converted to absorption spectra using the reciprocal relation (*Phys. Rev. B*, 2007, 76, (8), 085303).

$$EL(E) = EQE(E)\phi_{BB}(E)[\exp\left(\frac{qV}{kT}\right) - 1] \qquad \text{(eq. S5.1)}$$

Since the EL spectra of the donor-acceptor organic solar cells are dominated by the low energy CT state emission, the absorption spectra calculated from EL can be attached to the tails of the EQE spectra measured with the sensitive EQE setup. Complete EQE spectra spanning over 10 orders of magnitude are obtained for all of the solar cells investigated in this work, and the tails of the EQE spectra, corresponding to CT absorption, are fitted by the equation derived in the framework of Marcus theory (*J. Phys. Chem.*, 1989, 93, (8), 3078–3086), to determine the CT state properties.

$$EQE(E) = \frac{fE}{\sqrt{4\pi\lambda kT}}\exp(-\frac{(E_{CT}+\lambda-E)^2}{4\lambda kT}) \qquad \text{(eq. S5.2)}$$

To avoid an arbitrary fitting, the fit results for E_{CT} and λ are used to calculate the EL spectra of CT states, using:

$$EL(E) = \frac{fE^3}{\sqrt{4\pi\lambda kT}}\exp(-\frac{(E_{CT}-\lambda-E)^2}{4\lambda kT}) \qquad \text{(eq. S5.3)}$$

The calculated spectra agree well with the measured spectra for all of the solar cells studied in this work. The fit values for E_{CT} are then used to calculated ΔV_r, using the equation $\Delta V_r = E_{CT}/q - V_{OC,rad}$ (the method used to determine $V_{OC,rad}$ is described in SI-6): The calculated ΔV_r are compared to the lower limit for the radiative recombination voltage loss ($\Delta V_{r,sq}$) derived using the Shockley-Queisser theory, assuming that E_{CT} is the effective energy of bandgap for the solar cells, to verify that the fit results do not violate the theoretic boundary condition.

The EQE and EL spectra, and the fit results of the solar cells based on the DCDA polymers are given in Fig. 2 (Main text), and the results for the solar cells based on PBDB-T and PBDB-T(OD) mixed with different acceptors are shown in **Supplementary Fig. 17**. Note that Y6 and Y6(OD) are also used as the acceptors for the BHJ solar cells, however, due to the existence of strong S_1 state emission (*ACS Energy Lett.*, 2021, 6, (2), 557–564), it is difficult to determine E_{CT} from the sensitive EQE and EL spectra. Therefore, we use E_g instead of E_{CT} to determine the loss values. The E_g values are determined following the method described in the literature (*Sustainable Energy Fuels*, 2018, 2, 538), as shown in **Supplementary Fig. 18**.

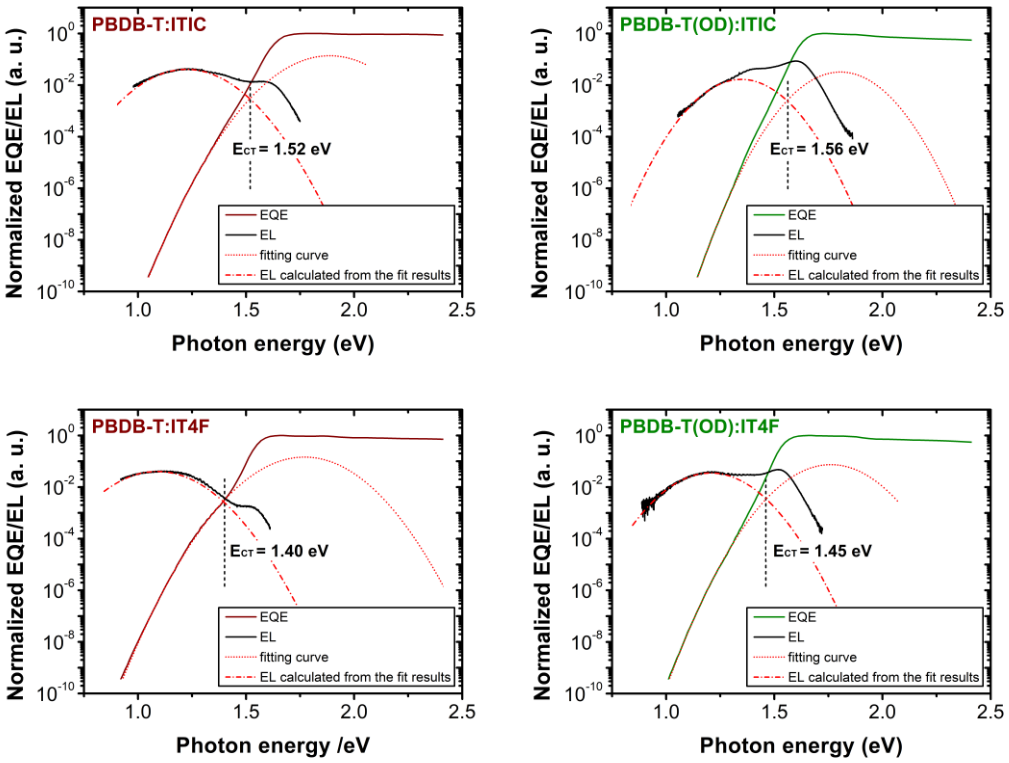

Supplementary Fig. 17. EQE and EL spectra of the BHJ solar cells based on PBDB-T and PBDB-T(OD) mixed with different acceptors.

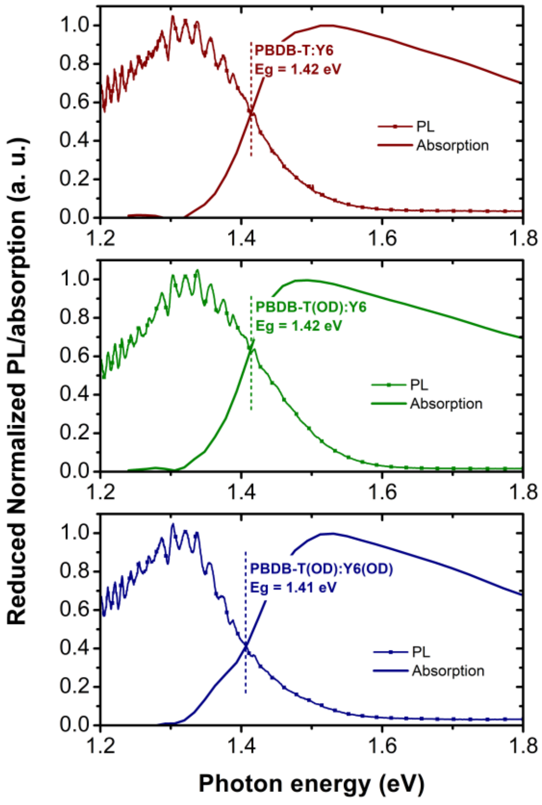

Supplementary Fig. 18. Reduced and normalized PL and absorption spectra of the BHJ films based on PBDB-T and PBDB-T(OD) mixed with Y6 and Y6(OD) for the determination of E_g.

Supplementary Note 6. Determination of ΔV_r and ΔV_{nr}

The voltage loss terms in the organic solar cells studied in this work are evaluated using two different methods. The first method relies on the measurement of the sensitive EQE spectra for the determination of $J_{0,rad}$, i.e., the radiative recombination limit for the dark saturation current,

$$J_{0,rad} = q \int EQE(E) \cdot BB(E) dE \qquad \text{(eq. S6.1)}$$

where $BB(E)$ is the blackbody emission photon flux and E is the photon energy.

$V_{OC,rad}$, the radiative limit for the V_{OC} of the solar cell (with 100% EQE_{EL}), is determined using the following equation.

$$V_{OC,rad} = \frac{kT}{q} \ln(\frac{J_{photo}}{J_{0,rad}}) \qquad \text{(eq. S6.2)}$$

where k is the Boltzmann constant, q is the elementary charge, and J_{photo} is the photocurrent density in the solar cell under an open-circuit voltage (assumed to be equivalent to the short-circuit current density).

ΔV_{nr} of the solar cell is then determined, using

$$\Delta V_{nr} = V_{OC,rad} - V_{OC} \qquad \text{(eq. S6.3)}$$

and ΔV_r is determined, according to

$$\Delta V_r = V_{loss} - \Delta V_{nr} \qquad \text{(eq. S6.4)}$$

where V_{loss} is expressed as

$$V_{loss} = E_g/q - V_{OC} \qquad \text{(eq. S6.5)}$$

or

$$V_{loss} = E_{CT}/q - V_{OC} \qquad \text{(eq. S6.6)}$$

V_{loss} in the first equation consists of radiative and non-radiative voltage losses associated with CT decay, as well as the voltage loss associated with the energetic difference between S1 and CT states. The V_{loss} in the second equation consists of only radiative and non-radiative voltage losses due to CT state recombination.

The second method for determining the voltage loss terms in the solar cells used in this work is based on the direct measurement of the CT state EQE_{EL}, since

$$\Delta V_{nr} = -\frac{kT}{q} \ln(EQE_{EL}) \qquad \text{(eq. S6.7)}$$

Then, ΔV_r can be calculated using equation S6.4. This method does not require measuring the weak CT state absorption and emission spectra. However, it is less suited for determining the CT state properties of the solar cell with the EL emission primarily from the pristine materials in the active layer. The loss values determined from both sensitive EQE and EQE_{EL} for the solar cells studied in this work are listed below in **Supplementary Table 2**: Similar voltage loss values are obtained, regardless of the method used.

From **Supplementary Table 2**, it is clear that ΔV_r is not much affected by the change of the DA spacing. This indicates that k_r does not significantly change with the change of the DA spacing, since ΔV_r is (logarithmically) dependent on k_r. Thus, the main reason for the significant increase in EQE_{EL}, and thus reduction in ΔV_{nr} in the solar cells with increased DA spacings is ascribed to the different k_{nr}. However, it should be noted that the result does not suggest that k_r is completely independent of the DA distance.

Supplementary Table 2. Voltage loss values from subgap EQE and EQE_{EL} measurements for the solar cells studied in this work.

Active Materials	V_{loss} (V)	Calculated from EQE		Calculated from EQE_{EL}		
		ΔV_{nr} (V)	ΔV_r (V)	EQE_{EL}	ΔV_{nr} (V)	ΔV_r (V)
**PBDB-T-NDI(C6)**	**0.82**	**0.56**	0.26	2.06×10^{-9}	**0.50**	0.32
**PBDB-T-NDI(C12)**	**0.73**	**0.49**	0.24	1.52×10^{-8}	**0.45**	0.28
**PBDB-T-NDI(C18)**	**0.70**	**0.44**	0.26	5.06×10^{-8}	**0.42**	0.28
**PBDB-T:IT4F**	**0.69**	**0.44**	0.25	7.54×10^{-8}	**0.41**	0.28
**PBDB-T(OD):IT4F**	**0.62**	**0.37**	0.25	1.24×10^{-6}	**0.34**	0.28
**PBDB-T:ITIC**	**0.62**	**0.34**	0.28	1.85×10^{-6}	**0.33**	0.29
**PBDB-T(OD):ITIC**	**0.57**	**0.28**	0.29	2.04×10^{-5}	**0.27**	0.30
**PBDB-T:Y6**	**0.75**	**0.37**	0.38	2.50×10^{-7}	**0.38**	0.37
**PBDB-T(OD):Y6**	**0.65**	**0.29**	0.36	2.04×10^{-5}	**0.27**	0.38
**PBDB-T(OD):Y6OD**	**0.55**	**0.21**	0.34	3.87×10^{-4}	**0.20**	0.35

Supplementary Note 7. Transient photovoltage decay measurements

In this work, transient photovoltage decay signals of the solar cells based on the DCDA polymers are measured to probe the lifetime of charge carriers, closely related to the non-radiative decay rate of CT states. The measurements are done under different bias illumination intensities. For each bias illumination intensity, a pulsed illumination is employed to generate the transient signal, and the decay time of the transient signal, representing the lifetime of free charge carriers, is determined by fitting with an exponential decay function.

The relationship between the life time of charge carriers and the lifetime of CT states has been discussed in the literature (*Annu. Rev. Phys. Chem*. 2016. 67(113), 33), and more recently, in (*J. Phys. Chem. C* 2021, 125, 15590). Here, this relationship is simplified using the three-state model (**Supplementary Fig. 19**), in which the lifetime of charge carriers (τ_{FC}) is determined by the decay (k_{CT}) and the dissociation rate (k_{diss}) of CT state, as well as the decay rate of free charge carriers (k_{FC}), i.e., the rate of free charge carriers falling into CT states.

$$\tau_{FC} = \frac{1}{k_{CT}N_{CTC}} + N(\frac{1}{k_{diss}N_{CTC}} + \frac{1}{k_{FC}np}) \qquad \text{(eq. S7.1)}$$

where *N* represents the average number of times that the CT state would dissociate and reform, before it decays to the ground state, N_{CTC} is the density of CT states, and *n* and *p* are the concentrations of free electrons and holes, respectively.

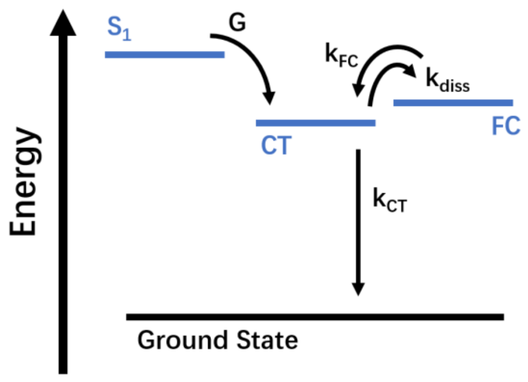

Supplementary Fig. 19. A three-state model describing the decay of free charge carriers (k_{FC}), the generation (*G*), dissociation (k_{diss}), and decay rate (k_{CT}) of CT states in a DA system.

Assuming that the generation rate of CT state is *G*, it can be derived (*Annu. Rev. Phys. Chem*. 2016. 67(113), 33):

$$N_{CTC} = \frac{G}{k_{CT}} \qquad \text{(eq. S7.2)}$$

$$k_{FC}np = \frac{k_{diss}G}{k_{CT}} \qquad \text{(eq. S7.3)}$$

Then, eq. S7.1 is reduced to

$$\tau_{FC} = \tau_{CT} + 2N\tau_{diss} \qquad \text{(eq. S7.4)}$$

where τ_{CT} is the lifetime of CT state, which equals to the inverse of the product of k_{CT} and N_{CTC}; and τ_{diss} is the time needed for the CT state to dissociate into free charge carriers, which is the inverse of the product of k_{diss} and N_{CTC}. Therefore, the key determining factor for τ_{FC} (voltage decay lifetime determined by the TPV measurement) is τ_{CT} and

τ_{diss}, and τ_{FC} linearly increases with τ_{CT}.

In **Supplementary Fig. 20**, the time constants of the solar cell based on PBDB-T-NDI(C18), are directly plotted as a function of the photovoltage (V_{ph}) generated by the bias illumination, and the V_{ph}s of the solar cell based on PBDB-T-NDI(C6) or PBDB-T-NDI(C12) are corrected by the difference in E_{CT} of PBDB-T-NDI(C18) and PBDB-T-NDI(C6), or PBDB-T-NDI(C18) and PBDB-T-NDI(C12). The correction of V_{ph} by the difference in E_{CT} allows us to compare the voltage decay lifetimes of the different solar cells with the same charge carrier density, assuming that the effective density of states in the active layers of the DCDA polymer based solar cells are similar. As shown in **Supplementary Fig. 20**, we find that the transient voltage decay time is considerably higher in the solar cell based on PBDB-T-NDI(C18), compared to the solar cell based on PBDB-T-NDI(C6) or PBDB-T-NDI(C12), suggesting that k_{nr} of the solar cell based on PBDB-T-NDI(C18) is much lower than that based on PBDB-T-NDI(C6) or PBDB-T-NDI(C12).

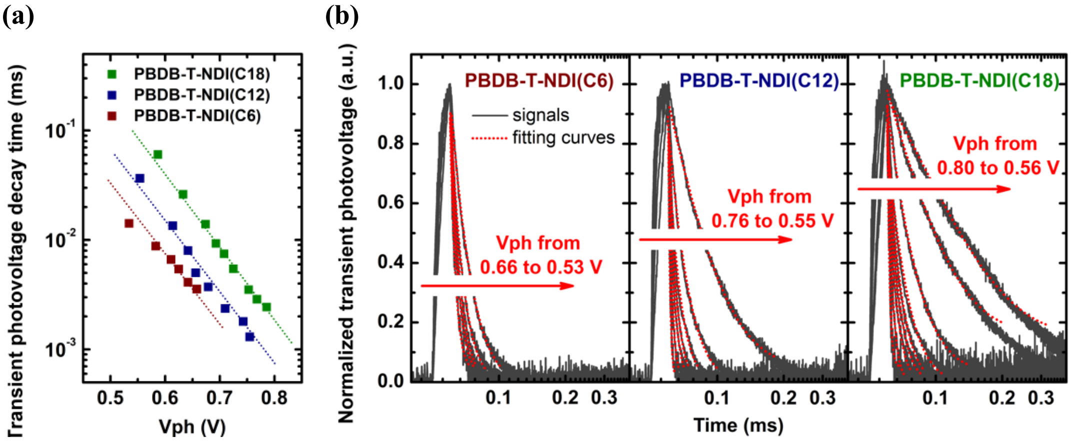

Supplementary Fig. 20. a) Transient photovoltage decay time as a function of V_{ph} (generated by the bias illumination) derived from the transient photovoltage measurements for the solar cells based on the DCDA polymers with different sizes of alkyl chain linkers. **b)** Transient voltage as a function of time for the solar cells based on the DCDA polymers measured under different illumination intensities.

The transient photovoltage decay times are also measured for the BHJ solar cells based on PBDB-T and PBDB-T(OD) mixed with different acceptors. The results for the systems based on Y6 and Y6(OD) are shown in Fig. 4 in the main text, and results for the systems based on ITIC and IT4F are shown below in **Supplementary Fig. 21**: We find that the transient voltage decay time is considerably higher in the solar cell based on PBDB-T(OD), compared to the solar cells based on PBDB-T, regardless of the acceptor used, suggesting that k_{nr} of the solar cells based on PBDB-T(OD) is lower than that based on PBDB-T.

(a)

(b)

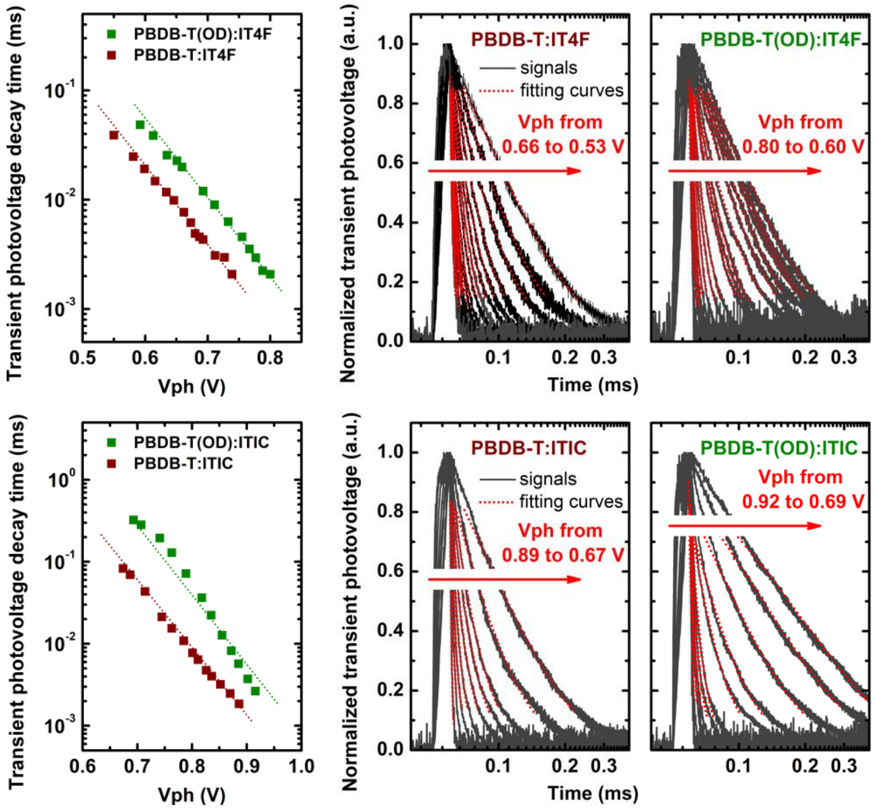

Supplementary Fig. 21. Transient photovoltage decay measurements for the BHJ solar cells based on PBDB-T and PBDB-T(OD) mixed with **a)** IT4F and **b)** ITIC.

Supplementary Note 8. Photoluminescence and external quantum efficiency

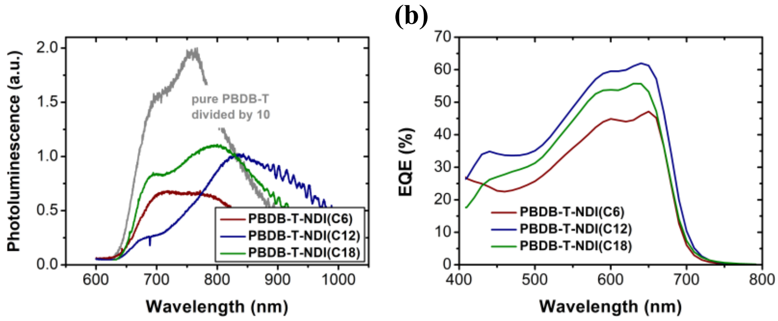

Supplementary Fig. 22. a) Photoluminescence spectra of the thin films based on the DCDA polymers with different sizes of alkyl chain linkers. The measurements are done using an excitation wavelength of 500 nm. **b)** EQE spectra of the solar cells based on the DCDA polymers.

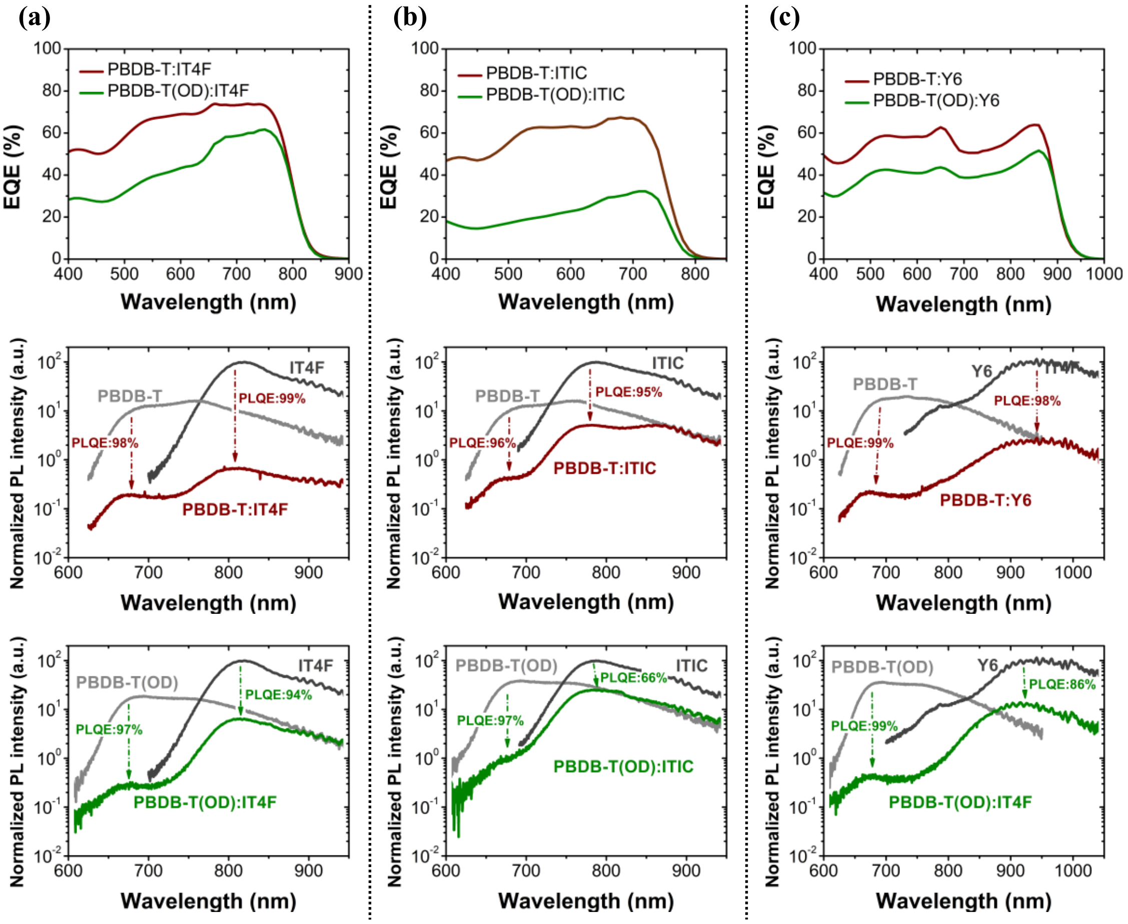

Supplementary Fig. 23. EQE spectra and photoluminescence quenching efficiency (PLQE) of the solar cells based on PBDB-T and PBDB-T(OD) mixed with **a)** IT4F, **b)** ITIC, and **c)** Y6.

Supplementary Note 9. JV curves of the solar cells

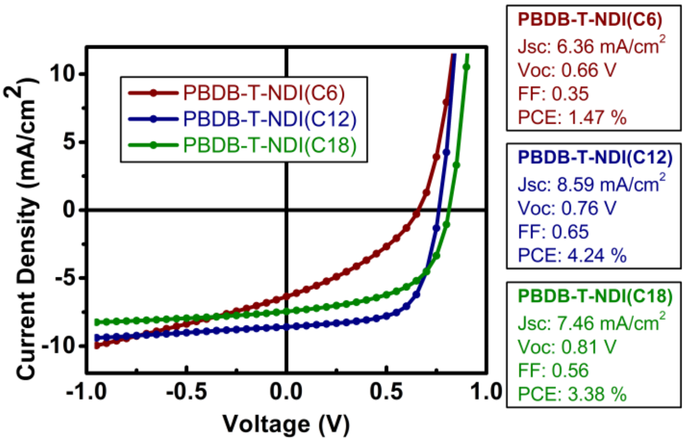

Supplementary Fig. 24. JV curves of the solar cells based on the DCDA polymers with different sizes of alkyl chain linkers.

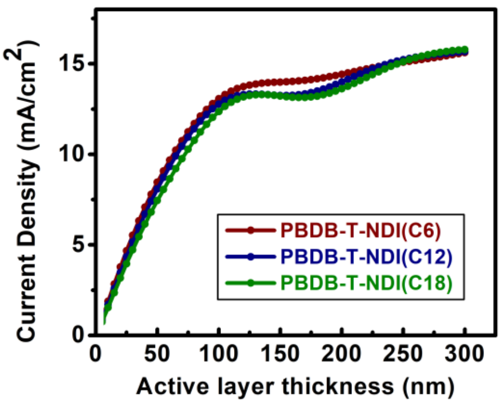

Supplementary Fig. 25. J_{sc} simulated by transfer matrix simulations for the DCDA solar cells with different active layer thicknesses. The optical constants of the materials used in the solar cells are measured by spectroscopic ellipsometry.

To understand the reason for the different J_{sc} of the solar cells based on the different DCDA polymers, transfer matrix model (TMM) simulations are performed, using the measured dielectric functions of the materials used in the solar cells (*J. Appl. Phys.* 1999, 86, 487). The results from the TMM simulations are shown in **Supplementary Fig. 25**.

We find that the J_{sc} predicted by the TMM are similar for all of the DCDA solar cells. This suggests that the difference in the measured J_{sc} of the solar cells is a result of different device internal quantum efficiencies (IQEs). For organic solar cells, IQE is primarily determined by the dissociation efficiency of the S_1 state into free charge carriers and their extraction efficiency. Since we found from the PL measurements (**Supplementary Fig. 22**) that the S_1 dissociation efficiency is high (over 90%) in all of the DCDA polymer based films, the lower IQE of the solar cell based on PBDB-T-NDI(C6) with the shortest linker, as compared to that based on PBDB-T-NDI(C12) and PBDB-T-NDI(C18), is most likely due to less efficient charge carrier extraction. This could also explain the strongly field dependent photocurrent extraction in the solar cell based on PBDB-T-NDI(C6) (**Supplementary Fig. 24**). In this work, we demonstrate that the decay rate of CT state is very high for the solar cell based on PBDB-T-NDI(C6), it is thus reasonable to attribute the rather inefficient photo-conversion to the high decay rate of the CT state, resulting in a comparably low IQE and low J_{sc} in the solar cell based on PBDB-T-NDI(C6).

For the solar cells based on PBDB-T-NDI(C12) and PBDB-T-NDI(C18), the extraction of photocurrent is independent of electric field (**Supplementary Fig. 24**). This suggests that in PBDB-T-NDI(C12) and PBDB-T-NDI(C18), the CT state decay rate, which is reduced as compared to that of PBDB-T-NDI(C6), does not limit the J_{sc}. Also, it suggests that the slightly lower J_{sc} of the solar cell based on PBDB-T-NDI(C18), as compared to that based on PBDB-T-NDI(C12), is due to less efficient dissociation of excitons. PL measurements (**Supplementary Fig. 23**) reveals that quenching of the acceptor emission is indeed less efficient in the active layer based on PBDB-T-NDI(C18), as compared to that based on PBDB-T-NDI(C12). This could be a result of a relatively higher degree of aggregation of the acceptor units in the film of PBDB-T-NDI(C18), due to the higher degree of spatial freedom of the acceptor units in the DCDA polymer with longer linkers.

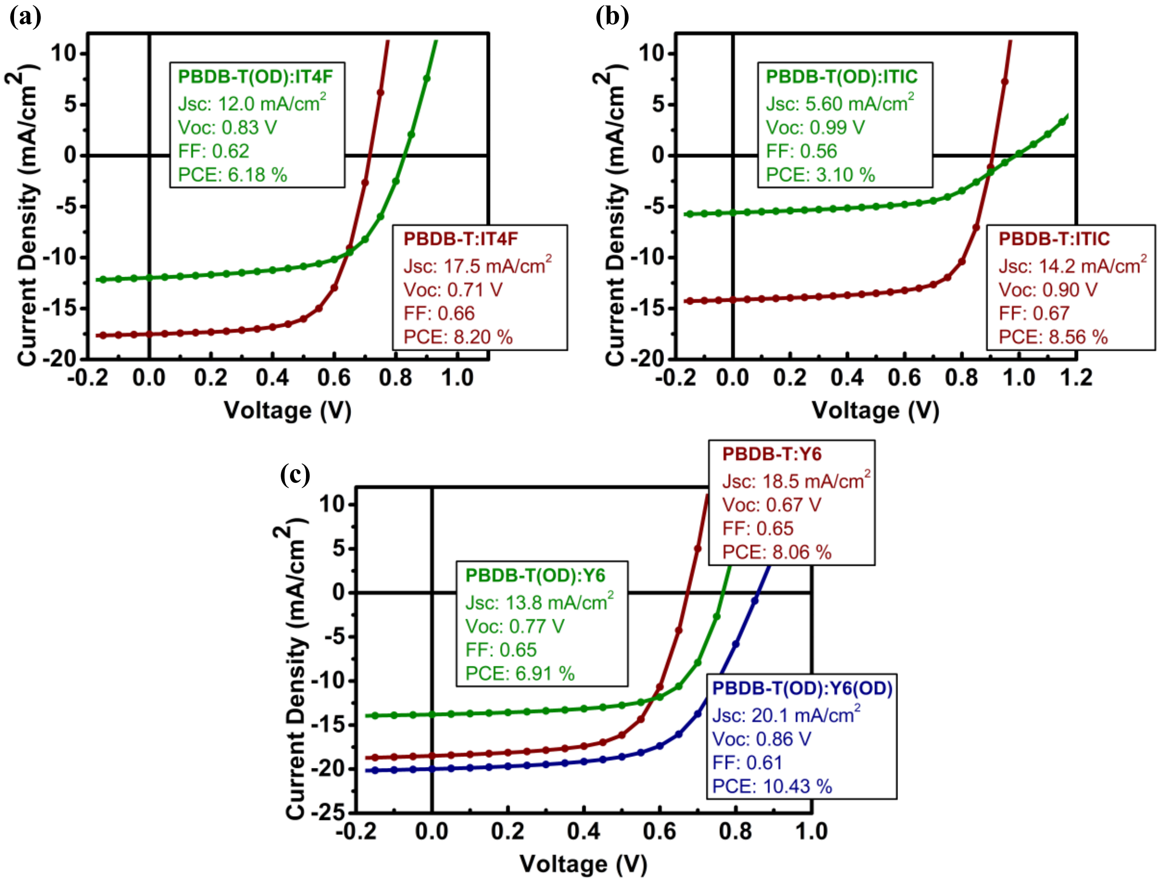

Supplementary Fig. 26. JV curves of the BHJ solar cells based on PBDB-T and PBDB-T(OD) mixed with **a)** IT4F, **b)** ITIC, and **c)** Y6 and Y6(OD).

Supplementary Methods

Materials and device fabrication:

Solar cells were fabricated on pre-patterned ITO substrates cleaned in an ultrasonicator, using detergent, acetone, isopropanol, and ethyl alcohol. Solar cells based on the DCDA polymers were constructed with an inverted architecture, i.e., ITO/ZnO/active layer/MoO_3/Ag. The ZnO interlayers were deposited using the sol-gel method reported in reference (*Advanced Materials* 2011, 23, (14), 1679): The precursor solution was prepared by mixing zinc acetate dihydrate (Aldrich, 99.9%, 1 g), dimethoxy ethanol (Aldrich, 99.8%, 10 mL), and ethanolamine (Aldrich, 99.5%, 0.28 g), and it was stirred at a speed of 1000 rpm, using a magnetic stirrer (IKA RCT digital) for 12 hours, prior to use. The precursor solution was deposited on the clean ITO substrates using a spin-coater (WS-650Mz-23NPPB, Laurell) with a speed of 3000 rpm, and then, dried at 200 °C on a hot plate (IKA RCT digital). The thickness of the ZnO layer was about 30 nm. The active layer solutions were prepared by dissolving the DCDA polymers in chlorobenzene (CB) (from Sigma-Aldrich). The concentrations of the solutions for PBDB-T-NDI(C6), PBDB-T-NDI(C12), and PBDB-T-NDI(C18) were 10 mg mL^{-1}, 12 mg mL^{-1}, and 12 mg mL^{-1}, respectively. The solutions were heated at 90 °C and stirred at a speed of 1000 rpm on a magnetic stirrer hotplate (IKA RCT digital) for 2 hours, prior to use. The active layers were spin-coated on the ZnO coated substrates at a speed of 1000 rpm in a glove box (Mbraun) filled with nitrogen, and they were annealed at 200 °C for 10 min on a hotplate (IKA RCT digital) before being transferred into a thermal evaporator (Mbraun), where a 7 nm thick MoO_3 layer and a 100 nm Ag layer were evaporated onto the active layer, under a vacuum pressure of 10^{-6} mbar, using a shadow mask. Finally, the devices were encapsulated using glass slides and a UV-curing glue. The thickness of the active layers was about 60 nm, and the active area of the solar cells was 0.04 cm^2, determined using an optical microscope.

The BHJ solar cells based on PBDB-T and PBDB-T(OD) mixed with different acceptors have a standard architecture, i.e., ITO/PEDOT:PSS 4083/active layer/PFN-Br/Ag. PEDOT:PSS 4083 (from Heraeus) was spin-coated on the clean ITO substrates, using a spin-coater (WS-650Mz-23NPPB, Laurell) at a speed of 5000 rpm. The thickness of the PEDOT:PSS layers was about 40 nm. Then, the PEDOT:PSS 4083 coated substrates were annealed at 150 °C for 20 min on a hotplate (IKA RCT digital). The active materials, PBDB-T, PBDB-T(OD), ITIC, IT4F, Y6, and Y6(OD) were purchased from Solarmer. The active layer solutions were prepared by dissolving the donor and the acceptor materials in CB mixed with 0.5% 1,8-diiodooctane (vol%). The donor:acceptor weight ratio for PBDB-T:IT4F, PBDB-T(OD):IT4F, PBDB-T:ITIC and PBDB-T(OD):ITIC was 1:1, and the ratio for PBDB-T:Y6, PBDB-T(OD):Y6, PBDB-T(OD):Y6(OD) was 1:1.2. The total concentrations of the solutions based on PBDB-T:IT4F, PBDB-T(OD):IT4F, PBDB-T:ITIC, PBDB-T(OD):ITIC, PBDB-T:Y6, PBDB-T(OD):Y6, and PBDB-T(OD):Y6(OD) were 20 mg mL^{-1}, 23 mg mL^{-1}, 20 mg mL^{-1}, 22 mg mL^{-1}, 21 mg mL^{-1}, 23 mg mL^{-1}, and 23 mg mL^{-1}, respectively. The active layer solutions were heated at 90 °C and stirred at a speed of 1000 rpm on a magnetic stirrer hotplate (IKA RCT digital) for 5 hours, prior to use. The active layers were spin-coated on the PEDOT:PSS coated substrates at a speed of 2500 rpm in a glove box (Mbraun) filled with nitrogen, and they were annealed for 10 min on a hotplate (IKA RCT digital). The annealing temperature was 120 °C for the active layers based on PBDB-T:ITIC and PBDB-T(OD):ITIC, it was 110 °C for the active layers based on PBDB-T:IT4F, PBDB-T(OD):IT4F, PBDB-T:Y6, PBDB-T(OD):Y6, and PBDB-T(OD):Y6(OD). PFN-Br used in this work was purchased from Sigma-Aldrich. The solvent for the PFN-Br solution was methyl alcohol, and the concentration of the solution was 0.5 mg mL^{-1}. The solution was spin-coated onto the BHJ active layers at a speed of 3000 rpm in the glovebox, and then transferred

into a thermal evaporator (Mbraun), where a 100 nm Ag layer was evaporated onto the active layer, under a vacuum pressure of 10^{-6} mbar, using a shadow mask. The thickness of the PFN-Br layers was about 1 nm, the thickness of the BHJ active layers was about 100 nm, and the active area of the solar cells was 0.04 cm^2.

Device characterization:

EQE spectra of the solar cells were measured using a halogen lamp (LSH-75, 250W, Newport), a monochromator (CS260-RG-3-MC-A, Newport), an optical chopper (3502 Optical Chopper, Newport), a front-end current amplifier (SR570, Stanford Instrument), and a lock-in amplifier (SR830, Stanford Instrument). A chopping frequency of 173 Hz was used for the measurements, and a set of optical filters (Newport) were used to eliminate the overtone wavelengths from the monochromator. A bias illumination of 100 mW cm^{-2} was used for the EQE measurements, and a focus lens and a mask were used to reduce the size of the illuminated area to about 0.5 mm^2.

J-V characteristics of the solar cells were determined by illuminating the solar cells with a solar simulator (Newport Oriel VeraSol-2™ Class AAA) with an illumination intensity of 100 mW cm^{-2}, calibrated using a standard silicon solar cell (calibrated by Hamamatsu Photonics) and a set of optical filters. The solar cells were placed in air (room temperature) during the measurements. The J-V curves were measured using a Keithley 2450 digital source meter. The measurements were done using a voltage step of 0.05 V, a dwell time of 1 s, a voltage scan range of -1 to 1.4 V. Over 10 individual devices were measured for each active material system, and an error of ± 0.01 V existed for the V_{OC} values. The J_{sc} values measured by the JV characterizations were slightly different from that derived from the EQE spectra (< 5%), and JV curves were corrected by the J_{sc}s calculated from the EQE measurements.

PL spectra of the organic layers were measured in dark environment using a Supercontinuous White Laser (SuperK EXU-6, NKT photonics) and narrowband filters (LLTF Contrast SR-VIS-HP8, LLTF Contrast SR-SWIR-HP8, NKT photonics) for a tunable excitation wavelength. The thin films were placed in front of the laser, and emission wavelengths of the thin films were collected by an optical fiber (BFL200LS02, Thorlab) connected to a fluorescence spectrometer (KYMERA-328I-B2, Andor Technology) with two sets of gratings for the visible and near-infrared wavelength range. The emission intensities were recorded by a Si EMCCD camera (DU970P-BVF, Andor Technology) for the wavelength range of 400~1000 nm, and an InGaAs camera (DU491A-1.7, Andor Technology), and the emission spectra were corrected for the optical losses, using a calibrated halogen lamp (HL-3P-CAL, Ocean Optics Germany GmbH).

EL spectra of the solar cells were measured using a source meter (Keithley 2400) for injecting electric current (1 mA), and the photons emitted from the solar cells were collected and recorded using the same optical fiber, spectrometer and cameras used for PL. The measured spectra were corrected for the optical losses in the fibers, the spectrometer, and the cameras.

EQE_{EL} of the solar cells were determined using a measurement system consists of a Keithley 2400 digital source meter, for injecting electric current into the solar cells, and a Keithley 6482 picoammeter for measuring the electric current from the Si photodiode used to collect the photons emitted from the solar cells.

Transient photovoltage measurements were done using a green LED, powered by a Keithley 2450 source meter, for the bias illumination and another green LED, powered by an arbitrary function generator (AFG3022C, Tektronix), for the pulsed illumination. The solar cells were placed in front of the LEDs, and voltage signals were recorded using an oscilloscope (MDO4104C, Tektronix). The intensity of the bias illumination was first set to generate a device photovoltage close to the V_{OC} of the solar cell under simulated AM1.5 illumination, and the intensity of the pulsed illumination was tuned to generate an additional transient photovoltage increasing the device voltage by about 5%. Then, the intensity of the bias illumination was gradually reduced, and the pulse illumination intensity was adjusted accordingly,

for the determination of the transient voltage decay times for solar cells under different bias illumination intensities.

Absorption spectra of the thin films were measured using a UV-Vis spectrometer (Lambda 950, PerkinElmer).

Thicknesses of the interlayers and active layers were determined using a profilometer (KLA-Tencor P-7 Stylus Profileror) or a Variable Angle Spectroscopic Ellipsometer (J. A. Woollam M-2000).

^{1}H-NMR and ^{13}C-NMR spectra were recorded at 400 MHz and 100 MHz on a Bruker AVANCE spectrometer with tetramethylsilane (TMS) as the internal standard. The Molecular weight was determined with gel permeation chromatography at 140 °C on a PL-GPC 220 system using a PL-GEL 13 μm Olexis column and *o*-DCB as the eluent against polystyrene standards. Cyclic voltammetry was performed by the CHI-660c electrochemical workstation system under an inert atmosphere at a scan rate of 0.1 V s^{-1} and 1 M tetrabutylammonium hexafluorophosphate in acetonitrile as the electrolyte, a glassy-carbon working electrode coated with samples, a platinum-wire auxiliary electrode, and an Ag/AgCl reference electrode.

Two-dimensional (2D) GIWAXS measurements were carried out on a GANESHA 300XL+ system from JJ X-ray in the X-ray lab at DSM Materials Sciences Center (DMSC). The instrument is equipped with a Pilatus 300 K detector, with pixel size of 172 μm × 172 μm. The X-ray source is a Genix 3D Microfocus Sealed Tube X-Ray Cu-source with an integrated Monochromator (multilayer optic "3D version" optimized for SAXS) (30 W). The wavelength used is λ = 1.5418 Å. The detector moves in a vacuum chamber with sample-to-detector distance varied between 0.115 m and 1.47 m depending on the configuration used, as calibrated using silver behenate (d_{001} = 58.380 Å). The minimized background scattering and the high-performance detector allow for a detection q-range varying from $3x10^{-3}$ to 3 $Å^{-1}$ (0.2 to 210 nm). The sample was placed vertically on the goniometer and tilted to a glancing angle of 0.2° with respect to the incoming beam. A small beam was used to get a high resolution. The primary slits have a size of 0.3 mm (horizontal) × 0.5 mm (vertical), and the guard slits have a size of 0.1 mm (horizontal) × 0.3(horizontal) mm. The accumulation time was 6 h for each measurement.
