# Supplementary material for: Increasing donor-acceptor spacing for reduced voltage loss in organic solar cells
Source: Nat Commun. 2021 Nov 18;12:6679. doi: 10.1038/s41467-021-26995-1 (PMC8602729; doi:10.1038/s41467-021-26995-1)
Supplement: Supplementary file 3 — Solar Cells Reporting Summary [file 41467_2021_26995_MOESM3_ESM.pdf]

nature research

Corresponding author(s): Weiwei Li, Zheng Tang, Koen Vandewal

Solar Cells Reporting Summary

Nature Research wishes to improve the reproducibility of the work that we publish. This form is intended for publication with all accepted papers reporting the characterization of photovoltaic devices and provides structure for consistency and transparency in reporting. Some list items might not apply to an individual manuscript, but all fields must be completed for clarity.

For further information on Nature Research policies, including our data availability policy, see Authors & Referees.

Experimental design

Please check: are the following details reported in the manuscript?

1. Dimensions

Item	Yes/No	Details
Area of the tested solar cells	☒ Yes ☐ No	Supporting Information
Method used to determine the device area	☒ Yes ☐ No	Supporting Information

2. Current-voltage characterization

Item	Yes/No	Details
Current density-voltage (J-V) plots in both forward and backward direction	☐ Yes ☒ No	No difference observed for the JV curves measured in forward and backward directions
Voltage scan conditions<br>*For instance: scan direction, speed, dwell times*	☒ Yes ☐ No	Supporting Information
Test environment<br>*For instance: characterization temperature, in air or in glove box*	☒ Yes ☐ No	Supporting Information
Protocol for preconditioning of the device before its characterization	☒ Yes ☐ No	Supporting Information
Stability of the J-V characteristic<br>*Verified with time evolution of the maximum power point or with the photocurrent at maximum power point; see ref. 7 for details.*	☐ Yes ☒ No	This work does not report high power conversion efficiency values, but focuses on the device operation mechanism.

3. Hysteresis or any other unusual behaviour

Item	Yes/No	Details
Description of the unusual behaviour observed during the characterization	☐ Yes ☒ No	No unusual behavior observed during the measurement
Related experimental data	☐ Yes ☒ No	No unusual behavior observed during the measurement

4. Efficiency

Item	Yes/No	Details
External quantum efficiency (EQE) or incident photons to current efficiency (IPCE)	☒ Yes ☐ No	Supporting Information
A comparison between the integrated response under the standard reference spectrum and the response measure under the simulator	☒ Yes ☐ No	Supporting Information
For tandem solar cells, the bias illumination and bias voltage used for each subcell	☐ Yes ☒ No	No tandem solar cells studied in this work

5. Calibration

Item	Yes/No	Details
Light source and reference cell or sensor used for the characterization	☒ Yes ☐ No	Supporting Information
Confirmation that the reference cell was calibrated and certified	☒ Yes ☐ No	Supporting Information

Calculation of spectral mismatch between the reference cell and the devices under test

- [x] Yes
- [ ] No

Supporting Information

6. Mask/aperture

Size of the mask/aperture used during testing

- [x] Yes
- [ ] No

Supporting Information

Variation of the measured short-circuit current density with the mask/aperture area

- [ ] Yes
- [x] No

The reported short-circuit current density values are corrected by the current density derived from the sensitive EQE measurements (measured with a bias illumination intensity of 100 mW cm-2).

7. Performance certification

Identity of the independent certification laboratory that confirmed the photovoltaic performance

- [ ] Yes
- [x] No

This work does not report high power conversion efficiency values, but focuses on the device operation mechanism.

A copy of any certificate(s)
Provide in Supplementary Information

- [ ] Yes
- [x] No

This work does not report high power conversion efficiency values, but focuses on the device operation mechanism.

8. Statistics

Number of solar cells tested

- [x] Yes
- [ ] No

Supporting Information

Statistical analysis of the device performance

- [x] Yes
- [ ] No

Supporting Information

9. Long-term stability analysis

Type of analysis, bias conditions and environmental conditions
For instance: illumination type, temperature, atmosphere humidity, encapsulation method, preconditioning temperature

- [ ] Yes
- [x] No

This work focuses on the device operation mechanism.
